# The SPOTLIGHT virtual audit tool: a valid and reliable tool to assess obesogenic characteristics of the built environment

**DOI:** 10.1186/1476-072X-13-52

**Published:** 2014-12-16

**Authors:** John R Bethlehem, Joreintje D Mackenbach, Maher Ben-Rebah, Sofie Compernolle, Ketevan Glonti, Helga Bárdos, Harry R Rutter, Hélène Charreire, Jean-Michel Oppert, Johannes Brug, Jeroen Lakerveld

**Affiliations:** Department of Epidemiology and Biostatistics, EMGO Institute for Health and Care Research, VU University Medical Center, Amsterdam, the Netherlands; University Paris 13, Equipe de Recherche en Epidémiologie Nutritionnelle (EREN), UMR U1153 Inserm/U1125, Centre de Recherche en Epidémiologie et Biostatistiques Sorbonne Paris Cité, Bobigny, France; Department of Movement and Sport Sciences, Faculty of Medicine and Health Sciences, Ghent University, Ghent, Belgium; European Centre on Health of Societies in Transition, London School of Hygiene and Tropical Medicine, London, UK; Department of Preventive Medicine, Faculty of Public Health, University of Debrecen, Debrecen, Hungary; Department of Nutrition, GH Pitié-Salpêtrière (AP-HP), University Pierre et Marie Curie-Paris6; Institute of Cardiometabolism and Nutrition (ICAN), Paris, France

**Keywords:** Virtual audit tool, Remote sensing techniques, Environmental characteristics, Reliability, Validity, Diet, Physical activity

## Abstract

**Background:**

A lack of physical activity and overconsumption of energy dense food is associated with overweight and obesity. The neighbourhood environment may stimulate or hinder the development and/or maintenance of a healthy lifestyle. To improve research on the obesogenicity of neighbourhood environments, reliable, valid and convenient assessment methods of potential obesogenic characteristics of neighbourhood environments are needed. This study examines the reliability and validity of the SPOTLIGHT-Virtual Audit Tool (S-VAT), which uses remote sensing techniques (Street View feature in Google Earth) for desk-based assessment of environmental obesogenicity.

**Methods:**

A total of 128 street segments in four Dutch urban neighbourhoods – heterogeneous in socio-economic status and residential density – were assessed using the S-VAT. Environmental characteristics were categorised as walking related items, cycling related items, public transport, aesthetics, land use-mix, grocery stores, food outlets and physical activity facilities. To assess concordance of inter- and intra-observer reliability of the Street View feature in Google Earth, and validity scores with real life audits, percentage agreement and Cohen's Kappa (k) were calculated.

**Results:**

Intra-observer reliability was high and ranged from 91.7% agreement (k = 0.654) to 100% agreement (k = 1.000) with an overall agreement of 96.4% (k = 0.848). Inter-observer reliability results ranged from substantial agreement 78.6% (k = 0.440) to high agreement, 99.2% (k = 0.579), with an overall agreement of 91.5% (k = 0.595). Criterion validity was substantial to high for most of the categories ranging from 87.3% agreement (k = 0.539) to 99.9% agreement (k = 0.887) with an overall score of 95.6% agreement (k = 0.747).

**Conclusion:**

These study results suggest that the S-VAT is a highly reliable and valid remote sensing tool to assess potential obesogenic environmental characteristics.

**Electronic supplementary material:**

The online version of this article (doi:10.1186/1476-072X-13-52) contains supplementary material, which is available to authorized users.

## Background

Obesity and overweight are recognised as important public health concerns and are often the result of an unhealthy lifestyle: a combination of insufficient physical activity and a long- term overconsumption of energy dense food [1–6]. According to socio-ecological models of health behaviour, characteristics of the physical environments in which we live (e.g. land use, street design, recreational facilities, presence and density of food outlets) substantially influence these unhealthy lifestyle behaviours and thus the likelihood of overweight and obesity [1–9]. To date, however, the evidence on the nature of this association of most physical environmental characteristics with unhealthy lifestyle behaviours and - especially - obesity is mixed and still unconvincing [4–6, 10, 11]. Very little evidence exists from studies that characterise environmental characteristics in an adequate and harmonised way *and* that provide consistent findings. For example, studies on fast food density and obesity use comparable measures but do not provide consistent results, while the operationalization of ‘land-use mix’ is more complex and provide relatively consistent evidence for an association with obesity [3]. This strengthens the belief that part of the inconsistent results may be due to inconsistent measurement and operationalization, and indicates the need for reliable and valid measures to assess obesogenic characteristics of neighbourhood environments. As such, there is a growing interest in enhanced ways to assess these kinds of environmental characteristics [2, 4, 5, 12].

Geographical Information Systems (GIS) methods could provide access to objectively characterised data on environmental characteristics [13–15] and may include data on food outlets from the Internet (websites of food chains), government sources or private sources [16]. However, as these data do not necessarily follow consistent standards for defining environmental characteristics and only rarely provide a high level of detail, field audits are often carried out [17, 18]. In a typical field audit, assessors walk a predetermined route through a specific area and use a scoring form to assess predefined environmental characteristics. In order to save time and resources, many researchers have advocated the use of remote sensing facilities such as Google Street View (GSV) or Bing Maps to perform desk-based assessments of environmental characteristics (4, 8, 14, 15-17-20). Other advantages of using remote sensing techniques for audits are that they can be performed anytime and anywhere, providing opportunities for large-scale neighbourhood audits without incurring the expense of travel or the risks of working in unsafe neighbourhoods [4, 8, 18–20]. Moreover, standardization and quality control can be better ensured. For example, compliance to assessment protocols can be more easily observed and documented as all data and imagery can be stored for reassessment. Since GE and GSV often provide street view imagery of different time periods, this may enable the analysis of environmental changes over time in longitudinal studies [4, 18, 19].

Remote sensing techniques are freely available through online applications such as GSV and Google Earth (GE) which make it possible to perform a ‘virtual audit’ and map entire neighbourhoods [4, 21]. GSV is currently the most commonly accessible form of 360 degree imagery at street level and provides good coverage of major cities around the world, making it a potentially useful tool for virtual analyses of neighbourhood environments [14, 19].

A number of studies used GSV to characterize environments [8, 14, 15, 17–28]. The majority of these studies were carried out in the United States, Australia or the United Kingdom, and mainly focused on physical-activity-related environmental characteristics [8, 14, 15, 17–24], determinants of communicable diseases [25, 28] or pollution [26, 27]. These studies indicated an accurate and consistent agreement between the interpretation of GSV data and regular field audits, suggesting that GSV is a valid medium for performing street audits [4]. Although geographic data on food outlets from other sources (such as Yellow Pages or government sources) could be combined with existing virtual audit tools, the use of a tool that captures different types of environmental factors would enable harmonised data collection in varying settings and across multiple countries. Additionally, performing a virtual audit in GE/GSV can be combined with the data collection of geographic locations of specific environmental elements, for instance, by simultaneously storing geolocations (coordinates) of places of interest. Such a comprehensive tool would therefore fill a niche for researchers aiming to map potentially obesogenic neighbourhoods, although the validity and reliability have not yet been determined.

We developed the SPOTLIGHT-Virtual Audit Tool (S-VAT) as part of the larger EU-funded SPOTLIGHT project [10] to assess the obesogenicity of neighbourhoods. This tool is based on items from validated virtual and field audit tools, and is the first tool to combine physical activity and food related environmental characteristics [12, 22, 24, 29–34]. The S-VAT has been developed to identify and compare environmental characteristics in European neighbourhoods using the GSV feature in GE. To assess the reliability and validity of this tool, this study aims to examine i) the inter-observer reliability, ii) the intra-observer reliability, and iii), the criterion validity by comparing the S-VAT to field audits.

## Methods

### Setting

In this study, four neighbourhoods that represented a variety in residential area density (RAD) and socio-economic status (SES) were selected for assessing the validity and reliability of the SPOTLIGHT-VAT. These four neighbourhoods were part of the 12 Dutch urban neighbourhoods located in the 'Randstad', selected for assessment of obesogenic environments in the broader SPOTLIGHT project [35, 36]. The urban agglomeration ‘Randstad’ encompasses the four largest Dutch cities and their surroundings in the West of the Netherlands. “Neighbourhoods” were defined according to administrative boundaries as made by the CBS (Statistics Netherlands, http://www.cbs.nl). Data on RAD were obtained from the Urban Atlas database [35]. This atlas is a GIS database distributed by the European Environmental Agency, based on a compilation of satellite photographs covering Europe and providing high resolution land use data [37]. Two classes of RAD were used: high and low residential area density (>80% and < 50% of areas covered by residential buildings, respectively). Socio-economic status data were based on median income data for each neighbourhood from the Netherlands census database [36]. Two classes of SES were used: high and low SES (corresponding to the first and third tertiles). The combination of the RAD and SES levels allowed the definition of four categories (High RAD/High SES, High RAD/Low SES, Low RAD/High SES and Low RAD/Low SES). For the broader SPOTLIGHT project, three neighbourhoods in each category were randomly chosen. In this validation study, we selected one neighbourhood from each category based on feasibility and proximity to the study site. Furthermore, we made sure that the raters did not conduct audits in the city they lived in.

### Development of the S-VAT

**Figure 1:**
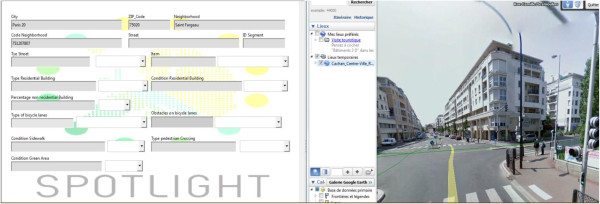
**Data input form in Open Office alongside GE.**

The S-VAT contains 40 different items and was based on previously published and validated tools [12, 22, 24, 29–34]. The tool was designed for the assessment of dietary and physical-activity-related environmental characteristics within neighbourhoods. The S-VAT was pilot-tested in 32 street segments, and refined accordingly. The final S-VAT incorporated items in eight main categories: walking related items (6 items), cycling related items (8 items), public transport (2 items), aesthetics (9 items), land use-mix (3 items), grocery stores (5 items), food outlets (6 items) and recreational facilities (3 items). The items 'type of street' and 'condition of sidewalk' are included twice in different categories. Each of the categories included multiple items, as depicted in Table 1. For a more detailed description of the individual items we refer to Additional files 1 and 2. A specific S-VAT data input form was created using Open Office open source software, with drop-down menu options for all responses (Figure 1). The data input form allows storage of images (screen capture) from GE. The form was designed to be viewed alongside the Street View feature in GE using a computer split-screen. To increase homogeneity between audits and to assist in clear and unambiguous scoring, a Standard Operating Procedure (SOP) was developed. The SOP describes information on the definition of street segments as well as the procedures for data extraction, data storage and defining environmental characteristics (Additional file 1).

**Table 1:** **Percentage agreement and Kappa statistics for all SPOTLIGHT-VAT items, presented by category**

Category	Inter observer reliability		Intra observer reliability		Criterion validity	
	% Agreement	Kappa	% Agreement	Kappa	% Agreement	Kappa
Presence of walking related items:*	**92.6**	**0.740**	**95.1**	**0.804**	**97.0**	**0.856**
Type of street (4 categories)	99.2	0.980	100	1.000	97.7	0.938
Presence of sidewalks (yes/no)	89.8	0.764	81.3	0.589	99.2	0.983
Condition of sidewalk (3 categories)	85.9	0.724	95.3	0.907	91.4	0.830
Pedestrian crossing available (yes/no)	95.3	0.642	96.9	0.761	96.9	0.761
Type of pedestrian crossing (if available)	94.5	0.589	96.9	0.765	96.9	0.766
Presence of streetlights (yes/no)	90.6	N/A**	100	N/A	100	N/A
Presence of cycling related items:*	**87.1**	**0.628**	**95.3**	**0.849**	**94.4**	**0.823**
Type of street (4 categories)	99.2	0.980	100	1.000	97.7	0.938
Presence of bicycle lanes (yes/no)	99.2	1.000	99.2	0.973	98.4	0.947
The speed limit in this segment	59.4	0.442	98.4	0.975	96.9	0.950
Obstacles present on bicycle lanes (yes/no)	89.1	0.085	93.8	0.531	95.3	0.557
Do cars form an obstacle on cycle lane (yes/no)	82.8	0.523	82.8	0.588	78.9	0.466
Traffic calming devices present (yes/no)	68.8	0.394	89.8	0.775	89.8	0.777
Public bicycle renting facilities (yes/no)	99.2	N/A	100	1.000	100	1.000
Type of bicycle lanes	99.2	0.973	98.4	0.948	98.4	0.949
Presence of public transport:*	**98.9**	**0.906**	**99.2**	**0.946**	**98.9**	**0.923**
Presence of bus/tram stop (yes/no)	97.7	0.811	98.4	0.892	97.7	0.845
Presence of railway/underground station (yes/no)	100	1.000	100	1.000	100	1.000
Aesthetics:*	**78.6**	**0.440**	**91.7**	**0.654**	**87.3**	**0.539**
Green/water area visible (yes/no)	69.5	0.393	90.6	0.793	88.3	0.740
Residential gardens visible (yes/no)	88.3	0.756	96.1	0.919	96.1	0.919
Rating of most residential buildings (3 categories)	85.2	0.416	93.8	0.796	92.2	0.769
Abandoned or vacant building/area visible (yes/no)	92.2	N/A	96.1	0.741	91.4	0.375
Maintenance of green areas (3 categories)	54.7	0.128	93.8	0.594	85.9	0.347
Condition of sidewalks (3 categories)	85.9	0.724	95.3	0.907	91.4	0.830
Presence of graffiti (yes/no)	90.6	0.306	89.1	0.590	82.8	0.454
Presence of litter (yes/no)	46.1	0.010	76.6	0.520	60.2	0.168
Presence of trees (yes/no)	95.3	0.786	93.8	0.698	97.7	0.883
Land use-mix:*	**80.1**	**0.285**	**91.4**	**0.762**	**91.1**	**0.740**
Presence of residential buildings (yes/no)	85.2	0.416	94.5	0.818	94.5	0.829
Type of residential buildings (5 categories)	72.7	0.000	89.1	0.836	90.6	0.861
% of non-residential buildings (5 categories)	82.5	0.440	90.5	0.633	88.1	0.531
Presence of grocery stores:*	**99.2**	**0.697**	**100**	**1.000**	**99.4**	**0.681**
Supermarket (yes/no)	98.4	N/A	100	1.000	99.2	0.663
Local food shop (yes/no)	97.7	0.393	100	1.000	99.2	0.886
Street food market (yes/no)	100	N/A	100	N/A	100	N/A
Wine/liquor store (yes/no)	100	N/A	100	N/A	100	N/A
Convenience store/small grocery store (yes/no)	100	1.000	100	1.000	98.4	0.494
Presence of food outlets:*	**99.2**	**0.579**	**99.7**	**0.887**	**99.9**	**0.887**
Restaurant (yes/no)	98.4	N/A	99.2	N/A	100	N/A
Fast food restaurant (yes/no)	98.4	0.494	99.2	0.663	99.2	0.663
Take away restaurant (yes/no)	99.2	N/A	100	1.000	100	1.000
On-street vendors of food (yes/no)	100	N/A	100	N/A	100	N/A
Café/bar (yes/no)	99.2	0.663	100	1.000	100	1.000
Shopping mall (yes/no)	100	N/A	100	N/A	100	N/A
Presence of physical activity facilities:*	**96.4**	**0.484**	**99.0**	**0.883**	**97.1**	**0.527**
Indoor recreational facility (yes/no)	100	1.000	100	1.000	99.2	0.663
Outdoor recreational facility (yes/no)	96.9	-0.012	100	1.000	96.9	0.317
Public park (yes/no)	92.2	0.465	96.9	0.650	95.3	0.602
Overall***	**91.5**	**0.595**	**96.4**	**0.848**	**95.6**	**0.747**

*Mean results of Percentage Agreement and Kappa for reliability and validity tests per category.

**N/A: not applicable.

***Mean results of Percentage Agreement and Kappa for reliability and validity tests summarized for all categories.

### Procedure

We assessed ten randomly chosen streets in each of the four Dutch neighbourhoods using the S-VAT. Comparability of streets was secured by dividing streets into segments. Street segments were defined as the part of the street between two intersections (with a minimum length of 50 meters and a maximum length of 300 meters) [14]. If streets crossed neighbourhood boundaries, they were assessed entirely or for 300 additional meters (when the street continued for more than 300 meters outside the neighbourhood boundary) [14]. The SOP for the virtual audit (Additional file 1) was adjusted in order to be suitable for the field audit (Additional file 2). Street View images are typically captured from a vehicle. Therefore areas prohibited for cars are generally less visible in Google Street View. Approximately five segments assessed in the current study were pedestrianized and not accessible to the Google car. However, because these segments were short and to a large extent visible from covered sections, we were able to rate these streets according to protocol.

The time taken to complete the field audit and the virtual audit was recorded. The same streets in each neighbourhood were selected to test the validity and reliability. As such, a total of 40 streets (128 street segments) were included in four neighbourhoods. Before the virtual audit took place, the assessors received training on using the S-VAT in the Street View feature of GE. To prevent bias, researchers did not conduct audits in the city in which they lived.

### Inter-observer reliability

The first researcher (JRB) performed virtual audits in the four selected neighbourhoods. To test the inter-observer reliability of the virtual audit, a second researcher (JDM) conducted virtual audits in the same streets per neighbourhood, independently from the first auditor. The two researchers were blinded to each other’s results.

### Intra-observer reliability

To test the reliability of the S-VAT over repeated audits, the virtual audit was conducted twice by the same auditor (JRB) to measure intra-observer variability. In order to reduce the effect of possible recall bias, the streets were audited in reverse order with at least a ten-day delay between the two audits.

### Criterion validity

In order to test the criterion validity of the S-VAT, the virtual audits were compared with field audits for 40 streets in total (ten per neighbourhood). The same data input form and codebook were used as in the virtual audit. The first researcher (JRB) conducted the virtual audit of the same street segments as the field audit, but in reversed order and with an interval of at least ten days between the field and virtual audit. Consequently, the researcher was less likely to be biased by his previous experiences of auditing those street segments.

### Statistical analysis

The inter/intra-observer reliability and criterion validity were measured by using Cohen’s Kappa (k). As low Kappa values can be seen despite reported high levels of agreement [14, 38], we also reported the proportions of agreement as described by de Vet et al. [38]. Kappa values of 0.80-1.00 were considered to represent high agreement, 0.60-0.79 as substantial agreement, 0.40-0.59 as moderate agreement, 0.20-0.39 as fair and 0.00-0.19 as slight agreement [39]. As sensitivity analysis, we explored whether validity and reliability across neighbourhood types. However, as shown in the Additional file 3: Table S1, there was not enough variability in items to calculate accurate validity and reliability statistics between neighbourhood types. All analyses were performed using SPSS (version 22).

## Results

The average time to conduct a virtual audit of one street segment was five minutes (range = 3–8 minutes), compared to an average time of ten minutes (range = 5–15 minutes) per street segment during the field audit. The prevalence of items per neighbourhood category are presented in Additional file 3: Table S1.

Table 1 provides an overview of the percentage agreement and Kappa statistics for the reliability and validity of separate items as well as for the overall category. The S-VAT showed substantial to high intra-observer reliability and criterion validity results, and moderate to high inter-observer reliability results for most of the street characteristics. Substantial to high percentage agreement was found for all results. Some Kappa values could not be calculated due to a lack of heterogeneity in responses.

### Intra-observer reliability

There was a high degree of conformity between the first and second virtual audit (96.4% overall agreement, k = 0.848), ranging from 91.7% agreement (k = 0.654) to 100% agreement (k = 1.000). When examining the results in more detail, the lower Kappa scores seen in *Aesthetics* mostly arose from variables such as 'Litter' (k = 0.520) and 'Graffiti' (k = 0.590).

### Inter-observer reliability

Inter-observer reliability results ranged from substantial (78.6% (k = 0.440)) to high agreement (99.2% (k = 0.579)), with an overall agreement of 91.5% (k = 0.595) between the two observers. Similar to the intra-observer reliability results, *Aesthetics* and *Land use-mix* were found to have the lowest Kappa scores (k = 0.440 and 0.285) and percentage agreement (78.6% and 80.1%). The items 'Litter' with 46% agreement (k = 0.010) and 'Maintenance of green areas' with 54.7% agreement (k = 0.128) resulted in poor agreement in the category *Aesthetics*. In the category *Land use-mix*, the item 'Type of residential buildings' with 72.7% agreement (k = 0.000) is responsible for the fair agreement. In contrast, high percentage agreement (98.9%) and Kappa (k = 0.906) were reported in the category *Public transport attractiveness.*

### Criterion validity

Agreement between the virtual and street audits was substantial to high for most of the categories ranging from 87.3% agreement (k = 0.539) to 99.9% agreement (k = 0.887) with an overall score of 95.6% agreement (k = 0.747). For *Aesthetics* and *Physical activity facilities*, agreement results were moderate. Variables such as 'Litter' (60.2% agreement, k = 0.168), 'Maintenance of green areas' (85.9% agreement, k = 0.347) and 'Outdoor recreational facilities' (96.9% agreement, k = 0.317) scored lowest. However, percentage agreement in all categories was found to be high (87.3% - 99.9%).

## Discussion

This study focused on validating the SPOTLIGHT-VAT, a virtual audit tool which was developed to map environmental characteristics related to physical activity and dietary behaviour. Our results showed that virtual audits based on the S-VAT are a valid and reliable way to assess neighbourhood characteristics that are potentially associated with physical activity and dietary behaviours. All environmental characteristics were reported to have substantial to high percentage agreement and moderate to high Kappa coefficients. This study supports findings from previous studies that remote sensing techniques like GSV and GE offer a reliable and valid alternative to field audits and are more time efficient [4, 14, 18–20, 22, 24, 40].

Our results demonstrate a good reliability and validity of food related environmental characteristics. As described by McKinnon et al. [41], the validity and reliability of tools to measure the food environment have not often been critically examined. A review on measurement of the food environment [42] describes that from the studies reporting on the psychometric properties of their instrument, tools to characterize food options in a community ‘generally report good to excellent reliability (Kappa around 0.70)’. Our results were comparable, with a slightly better reliability of ‘presence of grocery stores’ compared to ‘presence of food outlets’. This may be due to the fact that it is often easier to distinguish between a supermarket and a wine/liquor store than between a fast food restaurant and a take away restaurant.

In our study, we also observed substantial to high agreement in the categories *Walking related items* and *Cycling related items* in accordance with prior research [19, 24]. These findings are in contrast to Griew et al. [14] and Vanwolleghem et al. [23], who reported low to moderate agreement in these categories due to difficulties in defining the type and quality of sidewalks and cycle paths. High observed scores in the present study may be due to the fact that the audits were performed in the Netherlands, in which sidewalks and cycle paths are more often present, and of good overall quality.

Lowest agreement was found for items that require a subjective judgment from the auditor, such as 'Litter' and 'Graffiti' in the category *Aesthetics*. This is also consistent with previous literature describing lower levels of agreement for aesthetics and social characteristics [4, 9, 14, 18, 19, 22, 24, 40, 43]. This could be partially explained by temporal variability since GSV imagery was updated between the first virtual audit and the reliability tests, resulting in differences in imagery before and after the update. Alternatively, the lower reliability of 'Graffiti' and 'Litter' may be due to the fact that these items are more difficult to assess virtually due to obstructions like cars or trees that could block the view of the images [14, 19]. It may also indicate that personal perceptions between observers may play a role when assessing these types of items. The influence of personal perception was prevented as much as possible using a detailed SOP for the virtual audit (Additional file 1), as well as for the field audits (Additional file 2). However, to further increase inter-observer reliability, standardization and quality control measures (e.g. testing compliance to the assessment protocol) and setting specific examples are recommended when the audits are conducted by multiple observers [14, 19, 23].

### Strengths and limitations

Some potential limitations with regard to this study and the S-VAT should be noted. First, this study was conducted in Dutch neighbourhoods only. Therefore, generalization of results to environmental studies in other countries should be done with caution, since further inter country validation is needed. The tool however, appears particularly promising for performing inter country comparisons, using standardised methodology at lower costs and less time. Moreover, the use of different types of neighbourhoods (high SES, low SES, high RAD, low RAD) further increased the representativeness of the study.

Second, due to pragmatic reasons, the inter observer reliability and validity tests were conducted with only one other observer. Comparison with additional observers could strengthen the results. Third, there are some specific potential issues related to the temporal validity of GSV images [14, 17]. For instance, on-street vendors of food and obstacles present on the cycling lane are temporally variable and may or may not be present at the time the GSV vehicle drives by. Besides, we noted that GSV updated the imagery between the ratings of the first and second observer; this may have affected the inter rater reliability [22]. Also, the sometimes outdated images could have affected the criterion validity, as the oldest images dated from 2008 and the field audits were conducted in 2014. In the present study it was not feasible to assess differences between images taken over different years. However, Google is currently implementing the option to view images from several years. This may enable the assessment of changes in the physical environment over time. Finally, most GSV imagery was only available in areas accessible to cars. In areas where cars are prohibited, it is generally not possible to conduct virtual audits. Such pedestrianized areas may however, be particularly appealing for physical activity. Nevertheless, when the entrance of a car prohibited street was visible, all visible items were scored. As the street segments that were not accessible to the GSV car were often relatively short, all assessed segments in this study provided suitable data.

One of the strengths of this study is the relatively large number of different items assessed in the SPOTLIGHT-VAT in comparison to other tools [12, 22, 24, 29–33], resulting in a broad-based tool which is the first to assess neighbourhood environmental dietary and physical activity related characteristics. Also, the coverage of street segments in different neighbourhoods adds to the strength of the study, allowing a detailed view of neighbourhoods. What further adds to the strength of this research is that we were able to not only assess the criterion validity, but also the intra-observer reliability and inter-observer reliability. The use of percentage agreement in conjunction with Kappa may be seen as another strength since low Kappa coefficients may be reported despite high levels of agreement [14].

## Conclusion

The S-VAT is a valid and reliable tool to assess neighbourhood environmental characteristics associated with physical activity and eating behaviours that are potentially related to overweight and obesity. The tool is therefore fit to be used for future environmental research which is conducted with remote sensing techniques.

## Electronic supplementary material

Additional file 1: **SOP Virtual Audit.** Describes how the virtual audit is to be conducted. (DOC 13 MB)

Additional file 2: **SOP Field Audit.** Describes how the field audit is to be conducted. (DOC 8 MB)

Additional file 3: Table S1: Prevalence (%) of all SPOTLIGHT-VAT items per category, across different neighbourhood types and provides more detailed information on the study results. (DOC 120 KB)

## Abbreviations

CBS: Statistics Netherlands
GE: Google Earth
GIS: Geographical Information Systems
GSV: Google Street View
RAD: Residential Area Density
SES: Socio- Economic Status
SOP: Standard Operating Procedure
S-VAT: SPOTLIGHT Virtual Audit Tool.
