# Supplementary material for: The SPOTLIGHT virtual audit tool: a valid and reliable tool to assess obesogenic characteristics of the built environment
Source: Int J Health Geogr. 2014 Dec 16;13:52. doi: 10.1186/1476-072X-13-52 (PMC4279584; doi:10.1186/1476-072X-13-52)

**Additional file 2.**

**
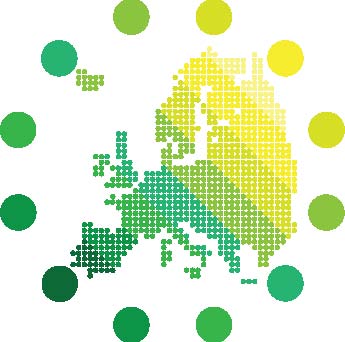
**

SPOTLIGHT

**The SPOTLIGHT virtual audit tool,**

**Standard Operation Procedure (SOP)**

**SPOTLIGHT WP3**: John Bethlehem, Helga Bardos, Johannes Brug, Helene Charreire, Sofie Compernolle, Ketevan Glonti, Jeroen Lakerveld, Joreintje Mackenbach, Jean-Michel Oppert, Maher Ben Rebah, Harry Rutter

**Content**

General Introduction 2

Preparation checklist 4

Conducting the virtual audit 4

Definition of street segments 9

Saving of items 12

Clarification of items 13

Appendix 1 21

Appendix 2 22

Appendix 3 23

Appendix 4 29

**General Introduction**

The SPOTLIGHT virtual audit tool (i.e. this SOP) is designed to assess key street-level features of the neighbourhood environment that are potentially related to physical activity and dietary behaviours - which are in turn related to obesity. This is only possible in countries/cities where Google Street View is available. SPOTLIGHT WP3 includes a sub-project which aims to assess actual environmental obesogenicity in selected neighbourhoods in five European Member States. Each Member State will individually assess their environmental obesogenicity. Therefore, the use of a standardised instrument will enable cross-country comparison of neighbourhoods for a common list of environmental characteristics. Environmental characteristics will be linked to health outcomes and lifestyle behaviours of inhabitants of the selected neighbourhoods.

The tool is based on a number of different existing tools (EPOCH 1, HAN, SPACES, ANC, PEDS, REAT, REIS, BESSC, Walkability audit tool and food environment classification tool). This tool assesses 8 general areas:

- Walking related items,
- Cycling related items,
- Public transport,
- Aesthetics,
- Land use-mix,
- Grocery stores,
- Food outlets,
- Physical activity facilities

Although much effort was put in this tool to be as comprehensive as possible, the tool is not complete in its assessment, which is an important aspect to bear in mind when analysing the results. Auditing neighbourhoods using this tool should be done using two computer-monitors and by a researcher/research assistant who has a background in health-related research and has received training in the use of the tool. It is essential that researchers familiarise themselves with all components of the tool before assessing the streets.

The SPOTLIGHT virtual audit tool (SPOTLIGHT-VAT) makes use of Google Street View (via Google Earth): online omnidirectional imagery of simultaneously collected images in multiple directions from a single location, thus producing a panoramic view. It allows the auditor to virtually walk through a neighbourhood to observe characteristics of the built environment. The SPOTLIGHT-VAT can be used in different places and at different time points, and allows for cross- country/city comparison of environmental characteristics. For some items (food/recreational-activity related items) it is necessary to pinpoint their exact geographical location. This can only be done via Google Earth. It is therefore necessary to download and install Google Earth in order to perform the virtual audit.

In this protocol, brief instructions will be given on ways how to conduct virtual audits of neighbourhood characteristics, as well as operational definitions for key concepts or terms that are not self-explanatory in the tool.

This SOP contains:

- a preparation checklist of resources needed during the audit,
- a step by step protocol on how to conduct the virtual audit,
- definitions of the area and street segments and how to save the audited items
- a clarification of the included items,
- appendix 1 and 2 show which push pins to use when defining street segments and food/recreational activity related items
- a more elaborate explanation of food related items can be found in appendix 3
- in appendix 4, brief instructions and tips on how to use Google Street View (in Google Earth) are provided.

**Preparation checklist**

***Doing a virtual audit checklist:***

- List of neighbourhoods to audit with clearly defined street segments,
- Copy of the SPOTLIGHT Virtual Audit Tool,
- Data entry file,
- Computer with two monitors,
- Computer mouse,
- Internet-access,
- Access to Microsoft Excel and Word,
- Access to Google Earth

**Conducting the virtual audit**

This audit is not based on predefined street segments. The auditor will define street segments en route (see chapter Definition of street segments) during the audit.

1. Open your internet browser
2. Start Google Earth by double clicking the icon. If Google Earth is not yet installed go to (http://www.google.com/earth/download/ge/agree.html) to download Google Earth and follow the instructions given by Google. When Google Earth is downloaded follow the installation instructions and start Google Earth
3. To navigate in Google Earth, follow provided instructions when starting Google Earth (also see Appendix 4 for tips and tricks)
4. Open the data entry file on the other screen. See the tab Definition of items for an explanation of the different items
5. Plan which streets you will be auditing in this session
6. Create your own folder in Google Earth in order to continue your own session the next time you open Google Earth. Go to Add in the upper task bar, click on Folder and type in the name of the folder. The name of the Folder should start with your first name continued by the name of the neighbourhood you are auditing (e.g. JohnBilthovenNoord). The name of your Folder will be shown in the Places tab on the left side of the screen (see example 1). Now, all items that will be saved, will be stored in your own Folder


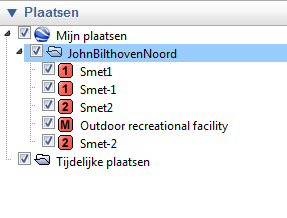


Example 1

1. Enter the postcode/street name in the Google Earth taskbar of the first street segment that you will be auditing and click on search. You will now be directed to the specific street you are about to audit
2. Start with the audit by defining the street segment (see chapter Definition of street segments for these instructions) before continuing to the next steps
3. Once the street segment is defined, start at the correct side of the street segment so that you don’t audit in the wrong direction and can continue to the next street segment more easily when finished. A helpful tool could be a detailed (printed out or Google maps) version of the streets in the neighbourhood so you know where the street starts and ends
4. Zoom in at street level by dragging the Pegman (the tiny yellow figure on the right side of the Google Earth map) to the right starting point on the map. This will start the Google Street View modus
5. Start with auditing on the left and right side of the street for about ten consecutive meters using the sequence of items given in the data entry file (this will be the approximate distance travelled when you scroll one step further in Google Street View)
6. Make sure that when filling in the data in the data entry file, each item is placed in the right cell so that data cannot be mixed or flawed, and make sure the right dropdown item is selected
7. When facing food outlets and recreational facilities, exact coordinates will be transcribed by using push pins (see Appendix 1). This can be done by following the steps written below. Each item will be given a specific push pin as to determine what type of item it is with the exact coordinates. The selected item will be placed between the starting and end point of the street segment (see example 2). This information may provide insight in the density of specific items within a neighbourhood


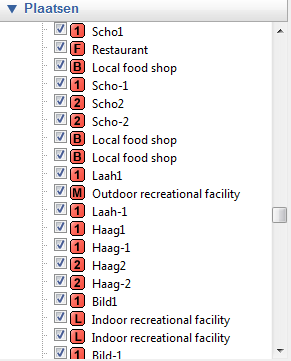


Example 2

1. Be sure that you only follow this procedure when facing a building on the front side (main entrance visible) in order to avoid assessing the same item twice
2. In order to give an exact coordinate to an item, go to the menu bar above the map and click on the yellow push pin icon
3. A menu similar to Example 3 will be opened and a yellow crosshair will be blinking on the map


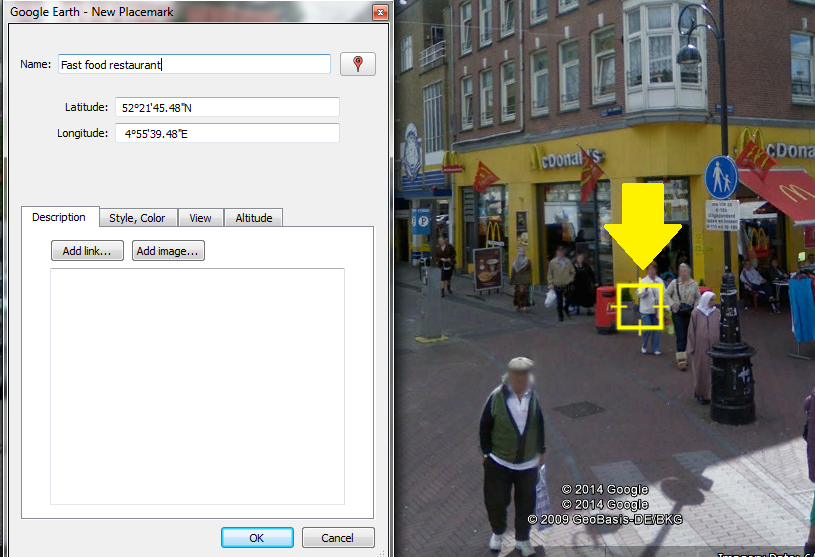


Example 3

1. Drag this yellow crosshair to the exact location that you want to transcribe (in the middle of an item)
2. Now you see the exact coordinates and a box called Name. Here you can fill in what kind of item this is
3. Click on the yellow push pin and select the type of push pin that belongs to the specific item (see Appendix 1 for an overview of the different options)
4. Click now on OK, now you can see that a description is given to your location (see example 2)
5. Make sure that the amount of food and recreational related items is given in the data entry file and the specific type of food item (see appendix 3)
6. Continue with auditing and repeat the process of saving coordinates of items when needed
7. When you are finished with auditing the first ten meters, mark a reference point for yourself when scrolling one click forward on your mouse so that you don’t forget as where you stopped with auditing. This reduces the risk of auditing the same characteristics twice or not at all (the distance between each click forward is approximately ten meters)
8. Click forward and repeat the previous steps
9. If necessary (e.g. when a lot of different items are visible), walk the segment twice or even three times to be sure you have filled out all the items
10. When finished with auditing a street segment, be sure to save your process (see chapter saving of items) before continuing to the next street segment
11. Count buildings when necessary and write down their ratings. In order to avoid double-counting different types of buildings, buildings should be counted on the segment with the building’s address or main entrance is visible.
12. After having rated and saved the first segment, continue to the second segment. Repeat this procedure until all segments of the street are assessed, before continuing to another street and/or neighbourhood

**Definition of street segments**

In this chapter instructions will be given on how to define street segments as is required in step 8 of the previous chapter.

1. Open Google Earth as described in the previously described steps (Google Earth is equipped with the latest maps of Google Street View, so maps are up-to-date)
2. Open the personalized folder as described in step 6 (conducting the virtual audit) in Google Earth in order to save the street segments
3. Enter the postcode/street name in the Google Earth taskbar of the first street that you will be defining and click on search. You will now be directed to the specific street
4. Zoom in at street level by dragging the Pegman (the tiny yellow figure on the right side of the Google Earth map) to the right starting point on the map. This will start the Google Street View modus. It is important to define street segments on street level since this is more detailed. When defining street segments from a ‘bird’s eye view’ (i.e. Google Earth) there is a chance you don’t see every intersection due to obstructions (e.g. trees or buildings) as demonstrated in example 4


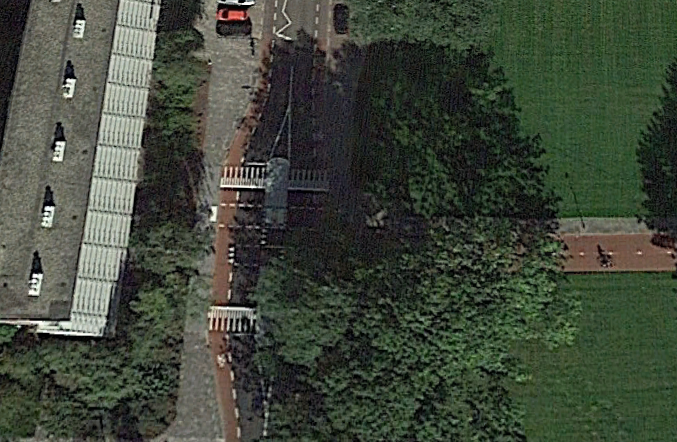


Example 4

1. Get to the starting point of the street (in the middle of the crossing, if possible, so you can clearly see the starting point of the street segment) you are about to define and at the correct side of the street, so that you don’t define the wrong segments and can continue to the next street segment more easily when finished. A helpful tool could be a detailed version of the streets in the neighbourhood or an overview of the neighbourhood via Google maps
2. By defining street segments push pins are designated to the start and ending point of each street segment (see Appendix 2 for the type of push pin required per segment)
3. Select the menu bar above the map and click on the yellow push pin icon to create the starting point of the street segment
4. A menu similar to example 5 will be opened and a yellow crosshair will be blinking on the map
5. Drag the yellow crosshair to the starting point of the segment (see example 5)
6. Type in the name of the street segment which will be the first four letters of the street name and the sequence number of the street segment. Be sure to give it the correct push pin (see Appendix 2) and click on OK. Now the street segment is saved and added to the


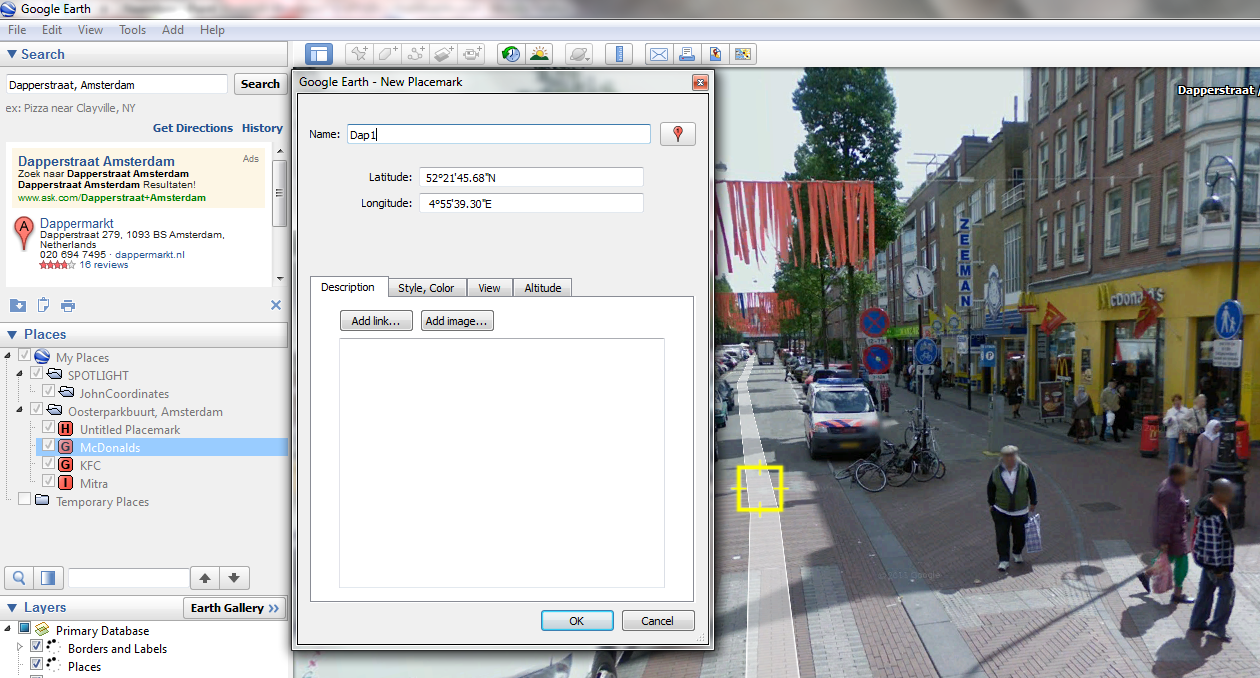


Example 5

1. When arriving at the end of the segment repeat the same procedure but then place the push pin at the beginning of the next crossing and give it a negative sequence number (e.g. Twen-1) to make clear which exact area is audited. This can sometimes be confusing when facing different crossings and/or alleys
2. Get to the beginning of the next street segment and repeat the same procedure (street segment 2, 3 etc.)
3. When naming of street segments is completed in a street, save your folder (see chapter Saving of items) and continue to the next street
4. When all street segments of the entire neighbourhood are defined, save your folder and create a new folder
5. Each street should be divided into street segments with a minimum length of 50 meters and a maximum length of 300 meters, between two intersections to maintain homogeneity of different street segments in long or short streets with few or many intersections
6. When segments are shorter than 50 meters but on a continuous part of the same street, segments are combined to one street segment
7. However, when segments are less than 50 meters and in a cul-de-sac, this segment should be defined as a separate street segment
8. Streets are audited entirely when reaching the end of the defined neighbourhood boundary, or for a 300 additional meters (when streets are longer)
9. Street segments are numbered so that different scored items can be better distinguished in the same street
10. The segments are made in Google Earth and saved as a KMZ-file (i.e. compatible with Google Earth) for longitudinal studies in order to assess differences in environmental characteristics over time

**Saving of items**

Create your own data entry file name using your own first name followed by DataEntryFile (e.g. JohnDataEntryFile). As described in chapter 'Conducting the virtual audit' you have to create your own Google Earth folder as well. In this way you can circumvent the risk that more people simultaneously work in the same file. When performing a virtual audit, it is important to immediately save the data entry file when completing each individual street segment. It is crucial that each street segment is assessed with the same amount of concentration, so be sure to take a break when you are feeling a loss of concentration.

When attributing coordinates to items and defining street segments in Google Earth, save your folder by clicking on Save Place As... First, click on your own folder so it is selected, then click on the File tab, go to Save and then to Save Place As and save as your own folder combined with the name of the audited neighbourhood (e.g. JohnBilthovenNoord) before continuing to the next street segment (see example 6). Repeat this process for each specific street segment. The file is saved as a KMZ-file (i.e. compatible with Google Earth) and named after the neighbourhood the street segments are in.


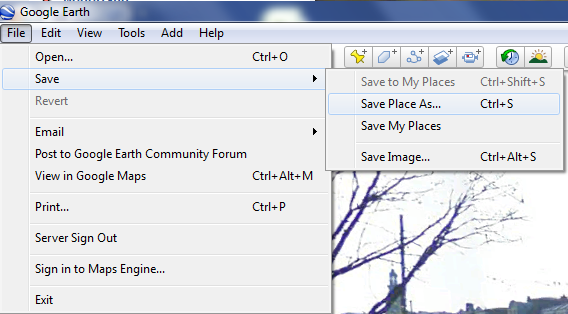


Example 6

**Clarification of items**

See appendix 3 for a detailed clarification of food related items.

| **Item** | **Category** | | | **Definition** | | |
| --- | --- | --- | --- | --- | --- | --- |
| **Walking related items** |  | | |  | | |
| Type of street: | Pedestrian friendly street | | | Area where motorized vehicles are allowed to drive max. 15 km/h. Road is shared with pedestrians, cyclists and cars. Children can play on the streets. This type of road can be an example of a 'woonerf' | | |
|  | Traffic sharing road | | | Road is mostly seen in residential or commercial area. Cars are allowed to drive, but need to give the right of way to cyclists and pedestrians. Maximum speed can differ | | |
|  | Regular road | | | Road in urban area where cars, cyclists and/or pedestrians have separated paths, with or without a buffer. Traffic speed does not exceed 50 km/h | | |
|  | Road with high-speed traffic | | | Main road that connects towns and cities, where speed exceeds 50 km/h. | | |
| Sidewalk present: | Yes/No | | | A man-made surface designed for pedestrians to use and can be only named sidewalk when it is associated with a roadway. | | |
| Sidewalk condition if sidewalk is present (see example 7) | Good | | | Good = no holes, sizable cracks, crumbling or uneven pavement on both sides of the street | | |
|  | Fair | | | Fair = holes, sizable cracks, or crumbling or uneven pavement along SOME of the side walk on both sides of the street | | |
|  | Poor | | | Poor = holes, sizable cracks, or crumbling or uneven pavement along most or ALL of the sidewalk on both sides of the street | | |
|  | Under construction | | | Under construction= (partially) blocked road, holes in road, construction work signage (only mark as under construction when there is a one sided sidewalk so that pedestrians have to walk on the road. When sidewalk on the other side of the street can be used, audit this part) | | |
| Pedestrian crossing available (see example 8) | Yes/No | | |  | | |
| Type of pedestrian crossing: | Zebra-path | | | Striped markings on the street, with or without signage for other road-users, but without traffic lights. | | |
|  | Over/underpass | | | Overpass is a bridge for pedestrians to cross over a street, underpass is a type of tunnel meant for pedestrians to cross under a street | | |
|  | Traffic lights | | | This can be any type of traffic lights meant for pedestrians to cross a street; automatic traffic lights, lights with pushbuttons etc. | | |
| Streetlights | Yes/No | | | This can be road- (for cyclists and cars) or pedestrian-oriented lighting and is present (at least two street lights) on a continuous stretch of road | | |
| 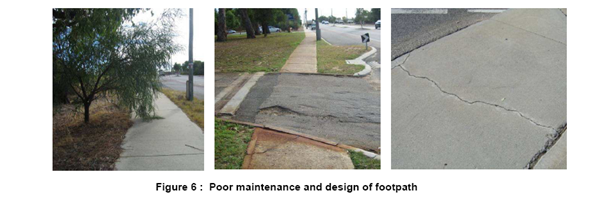  Example 7  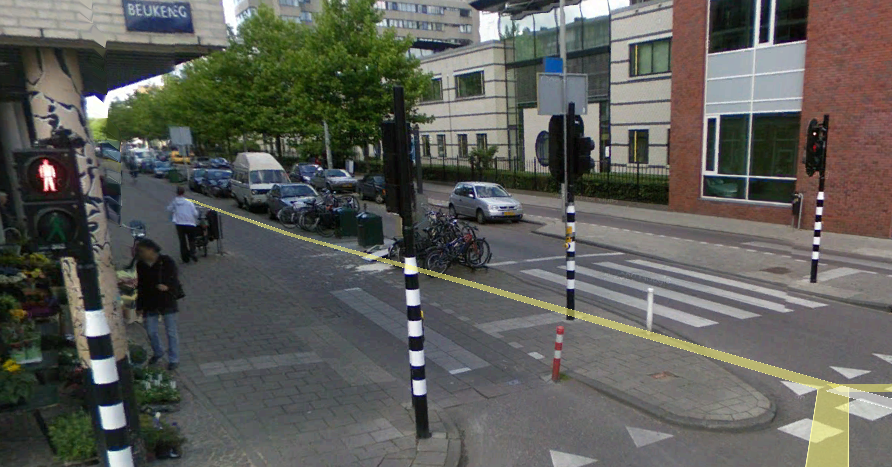  Example 8 | | | | | | |
| **Cycling related items** | | |  | |  | |
| Type of street: | | | Pedestrian friendly street | | Area where motorized vehicles are allowed to drive max. 15 km/h. Road is shared with pedestrians, cyclists and cars. Children can play on the streets. This type of road can be an example of a 'woonerf' | |
|  | | | Traffic sharing road | | Road is mostly seen in residential or commercial area. Cars are allowed to drive, but need to give the right of way to cyclists and pedestrians. Maximum speed can differ | |
|  | | | Regular road | | Road in urban area where cars, cyclists and/or pedestrians have separated paths, with or without a buffer. Traffic speed does not exceed 50 km/h | |
|  | | | Road with high-speed traffic | | Main road that connects towns and cities, where speed exceeds 50 km/h. | |
| Bicycle lane | | | Yes/No | | Lane on street where cyclists are allowed to cycle. This lane can or cannot be shared with other road-users | |
| What is the speed limit | | | Open space to write down speed | | Look at road speed signs on side of the street or on the road | |
| Obstacles present on bicycle lanes or place on road which is used by cyclists | | | Temporary | | This could be motorized vehicles, construction work, markets or any other objects that block the cycle path for a temporal time period. | |
|  | | | Permanent | | This could be a fence, building or any other obstacle which blocks the cycle path for a permanent time | |
|  | | | No | | No obstacles present which enables the cyclist to continue its path | |
| Cars form obstacles on road | | | Yes/No | | Cars parked on the road and/or partly on the sidewalk regardless of whether this is done legally or illegally. If cars are parked on the sidewalk and/or cycle path and cyclists and/or pedestrians have to manoeuvre around these cars, they form an obstacle | |
| Traffic calming devices | | | Yes/No | | Speed humps, traffic island, roundabouts, traffic lights | |
| Public bicycle facilities | | | Yes/No | | Facilities where bicycles can be rented. These facilities can be seen at for example railway-stations | |
| Type of bicycle lanes (see example 9) | | | On road cycle lane with markings | | Path used by cyclists, with centre line, logos and other markings included for safety or convenience | |
|  | | | Separate cycle lane with buffer | | Cycle lane is not shared with any other road-users and is separated from the street/sidewalk by a buffer and is specifically meant for cyclists | |
|  | | | Shared path with pedestrians | | Path used by pedestrians as well as cyclists, with or without markings | |
| 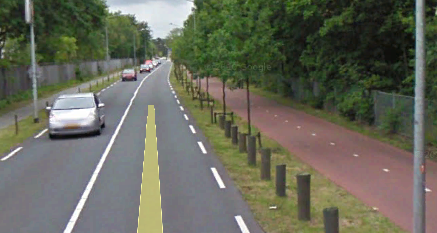 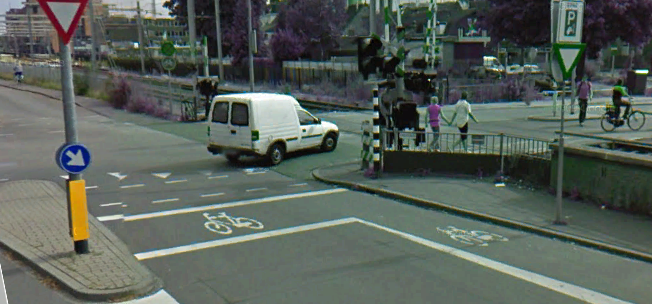  Separate cycle lane On road cycle lane with markings  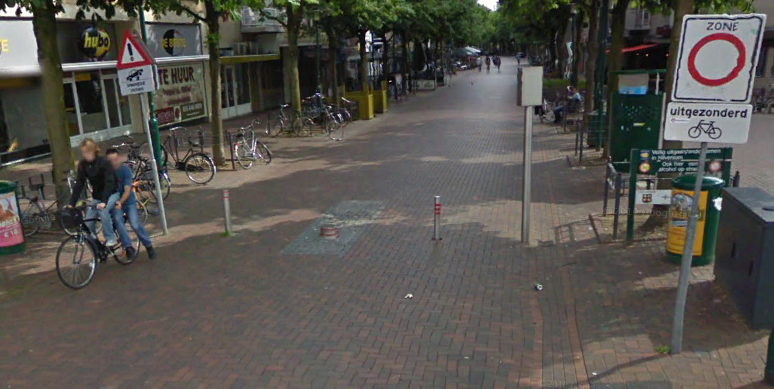  Shared path with pedestrians  Example 9   | **Public transport** |  |  | | --- | --- | --- | | Bus/tram stop | Yes/No | Presence of tram/bus transit stop where people have to wait in order to use the public transport facilities. Recognizable by signage, shelter or shoulder on road where bus/tram stops | | Railway/underground station | Yes/No | Presence of train/underground railway station. Recognizable by signage, train station | | | | | | | |
| **Aesthetics** | |  | | | |  |
| Green and water area visible | | Yes/No | | | | This can be any lake, river, park, forest or any other natural environmental surrounding |
| Residential gardens | | Yes/No | | | | Visibility of any residential gardens within street segment. If there is only a glimpse visible of a garden, then this garden would not be rated |
| Rating of condition of most residential buildings | | Well kept condition | | | | Housing looks well maintained, paint looks to be in good condition, gardens are taken care of |
|  | | Fair condition | | | | Housing is not well maintained but also not broken |
|  | | Poor/bad/detoriated condition | | | | Housing is not well maintained and broken windows, abandoned buildings are visible, gardens are not maintained |
| Abandoned building or vacant area | | Yes/No | | | | Building or area which is not being used. Only one vacant apartment should not be rated. When 50% or more of the apartment building is vacant then rate as abandoned. Only rate when it can be made clear that building houses no residents |
| Maintenance of green areas: | | Well maintained | | | | Verges, trees in residential gardens and/or public space, planted vegetation looks trimmed and clean |
|  | | Not well maintained | | | |  |
| Sidewalk condition if sidewalk is present (see example 7) | | Good | | | | Good = no holes, sizable cracks, crumbling or uneven pavement on both sides of the street |
|  | | Fair | | | | Fair = holes, sizable cracks, or crumbling or uneven pavement along SOME of the side walk on both sides of the street |
|  | | Poor | | | | Poor = holes, sizable cracks, or crumbling or uneven pavement along most or ALL of the sidewalk on both sides of the street |
|  | | Under construction | | | | Under construction= (partially) blocked road, holes in road, construction work signage (only mark as under construction when there is a one sided sidewalk so that pedestrians have to walk on the road. When sidewalk on the other side of the street can be used, audit this part) |
| Graffiti | | Yes/No | | | | Graffiti is defined as tags, and any other paint markings which could not be seen as street art which is placed on any buildings and or objects placed in the street |
| Litter | | Yes/No | | | | Trash visible on the streets, this can be a various range of discarded items like plastic bags, wrappings, cigarette packages and other discarded items laying on the street. Three or more discarded items on the streets can be scored as litter |
| Trees | | Yes/No | | | | Trees standing within this street segment |
|  | |  | | | |  |
| **Land use-mix** | |  | | | |  |
| Residential buildings visible | | Yes/No | | | | Housing where people only live in. Residential buildings need to be connected to the street where the audit is taking place. Front entrance or driveway must be connected to the street |
| Type of residential buildings: | | Detached/semidetached homes | | | | A house which is, or is not attached to another single house |
|  | | Terraced homes | | | | Multiple houses attached to each other |
|  | | Apartment buildings (<5 stories) | | | | Housing with apartments above and next to each other but the building is lower than (or equal to) 5 stories |
|  | | Apartment building (>5 stories) | | | | Housing with apartments above and next to each other but the building (including possible shops) is higher than 5 stories |
|  | | Apartment above shops | | | | Housing with one or more apartments above a shop |
| What is the percentage of non-residential buildings in comparison with residential buildings in this street segment | | 0% | | | | 0%= no residential buildings visible (rate 0% when there is only one or more non-residential buildings which in total are closer to 0% than 25%) , 25% = less non-residential buildings than residential buildings, 50% = about even distribution, 75%= more non-residential buildings than residential buildings, 100%= almost only non-residential buildings visible |
|  | | 25% | | | |  |
|  | | 50% | | | |  |
|  | | 75% | | | |  |
|  | | 100% | | | |  |
| **Grocery stores** | |  | | | |  |
| Supermarket | | Number and coordinates: | | | | Shop from an international or national chain where groceries/food supplies can be bought |
| Local food shop | | Number and coordinates: | | | | This can be a bakery, fish-shop, butcher, greengrocer |
| Street food market | | Number and coordinates: | | | | A vendor which sells food on a outdoor market. This can be fast-food or non-fast-food |
| Wine/liquor store | | Number and coordinates: | | | | Primarily sells alcohol containing drinks |
| Convenience store/small grocery store | | Number and coordinates: | | | | Smaller version of a supermarket but is from a local chain or from a gas-station company and can have different opening hours than larger supermarkets |
|  | |  | | | |  |
| **Food outlets** | |  | | | |  |
| Restaurant | | Number and coordinates: | | | | Non-fast-food restaurant, where people sit down to eat |
| Fast food restaurant | | Number and coordinates: | | | | This can be a international or local chain and is a destination that primarily sells carry-out burgers, fried chicken, sushi or other fast prepared foods (e.g. McDonalds, Döner kebab, KFC) |
| Take away restaurant | | Number and coordinates: | | | | Only rate when it is visible on the outside of the restaurant that this is a take away restaurant. This is the type of restaurant where people do not sit-down to eat but take their prepared meals elsewhere. See checklist food items for examples. |
| On-street vendors of food | | Number and coordinates: | | | | A vendor which sells fast-food on the street, like in a park or public square and sells hotdogs, spring-rolls or other snacks |
| Café/bar | | Number and coordinates: | | | | A place where people can drink coffee or other beverages with a small snack |
| Shopping mall | | Number and coordinates: | | | | Multiple shops housed in linked buildings |

| **Physical activity facilities** |  |  |
| --- | --- | --- |
| Indoor recreational facilities | Number and coordinates: | An indoor environment where people can exercise sports, or in any other way recreate. For example a gym, swimming pool or sports hall |
| Outdoor recreational facilities | Number and coordinates: | A man-made or natural outdoor environment where people can exercise sports, or in any other way recreate. For example a park, outdoor fitness area or skate park |
| Public park | Yes/No | Natural recreation area which is maintained and publicly accessible |

**Appendix 1**

**Selection of push pins in Google Earth**

| ***Food outlets, Grocery stores*** | ***Type of push pin*** |
| --- | --- |
| Supermarket | 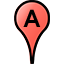 |
| Local food shop | 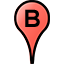 |
| Street food market | 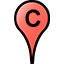 |
| On-street vendors of food | 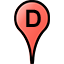 |
| Convenience store/small grocery store | 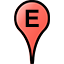 |
| Restaurant | 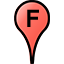 |
| Fast food restaurant | 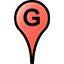 |
| Take away restaurant | 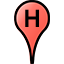 |
| Wine/liquor store | 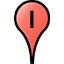 |
| Café/bar | 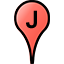 |
| Shopping mall | 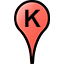 |
| Indoor recreational facilities | 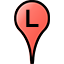 |
| Outdoor recreational facilities | 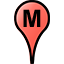 |
| Public park | 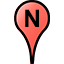 |

**Appendix 2**

| ***Street Segment*** | ***Type of push pin*** |
| --- | --- |
| 1 | 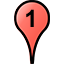 |
| 2 | 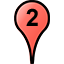 |
| 3 | 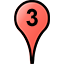 |
| 4 | 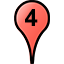 |
| 5 | 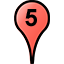 |
| 6 | 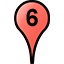 |
| 7 | 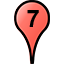 |
| 8 | 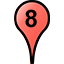 |
| 9 | 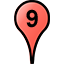 |
| 10 | 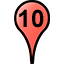 |

**Appendix 3**

**Food environment categories**

1. Supermarket 7
2. Local shops 5 and 9
3. Street food market 10
4. On street vendors 10
5. Convenience stores 8 and 7.3
6. Restaurants 1
7. Fast food restaurant 6.4
8. Take-away restaurant 6 and 4
9. Wine & liquor stores 8.4
10. Café-bar 2 and 3
11. Shopping mall (not in the list)

| **1. Restaurant, Pub & Hotel Restaurant** | | |
| --- | --- | --- |
| Code | Type of food outlet | Description |
| 1.1 | Traditional | Sit down restaurant |
|  |  | Waiter/waitress takes your order |
|  |  | Pay for meal after eating |
| 1.2 | Buffet | Sit down restaurant |
|  |  | No waiter service |
|  |  | May pay at the till after food has been selected from the buffet but before eating |
|  |  | If 'all you can eat' at a fixed price may pay before or after consumption. Drinks may or may not be included in the price. |
| 1.3 | Restaurant with takeaway/delivery option | Primarily a restaurant but has the option to order for takeout |
|  |  | Waitress/ waiter service or food is ordered and paid for at the counter and eaten elsewhere |
|  |  | Usually open after 5pm |
|  |  | Examples include Chinese restaurants, Indian restaurants, pizza hut |
| 1.4 | Fast casual (e.g. Nandos) | Order and pay for food at counter |
|  |  | Waiter/ waitress delivers food to table |
|  |  |  |
|  |  | Usually sit down but may have takeaway option |
| 1.5 | Pub sit down restaurant | Sells predominantly alcohol |
|  |  | Sit down restaurant |
|  |  | Waiter/ waitress takes your order |
|  |  | Pay for meal after eating |
| 1.6 | Pub fast casual | Sells predominantly alcohol |
|  |  | Order and pay for food at the bar. |
|  |  | Waiter/ waitress delivers food to table |
|  |  | Similar to fast food but offers a higher quality of food and atmosphere |
|  |  | Sit down only not takeaway |
| 1.7 | Pub with takeaway/ delivery option | Primarily a pub but has the option to order for takeout |
|  |  | Waitress/ waiter service or food is ordered and paid for at the counter and eaten elsewhere |
| 1.8 | Traditional hotel | Restaurant with waiter service |
|  |  | Room service and banqueting rooms |
|  |  | May have a buffet for selected meals (e.g. breakfast) |
|  |  | Light bar meals with/without waiter service |

| **2. Pub no food** | | |
| --- | --- | --- |
| 2.1 | Pub no food | Only alcoholic and non-alcoholic drinks served |
|  |  | May served crisps and nuts behind the bar |
|  |  | Included nightclubs |

| **3. Sit in café/coffee, specialist and sandwich shop** | | |
| --- | --- | --- |
| Code | Type of food outlet | Description |
| 3.1 | Traditional café | Predominantly coffee and hot beverages sold |
|  |  | Informal seating area |
|  |  | May have waiter service or order at the counter |
|  |  | Pre-made/ made to order sandwiches and confectionery available |
| 3.2 | Greasy spoon types café | Predominantly less healthy fried foods |
|  |  | Informal seating area |
|  |  | May have waiter service or order at the counter |
| 3.3 | Specialist café | Includes milkshakes/smoothie bars and ice cream shops |
|  |  | Similar in style to cafes and coffee shops |
|  |  | Informal seating area |
|  |  | Fair trade cafes/coffee shops are included here |
| 3.4 | Café with delicatessen/bakery | Predominantly café with delicatessen/bakery counter enabling ready-to-eat foods to be taken away |
|  |  | Informal seating area |
| 3.5 | Sit-in sandwich shop | Small seating area |
|  |  | Order and pay at the counter |
|  |  | Made to order sandwiches/salads etc. May sell drinks, branded snacks and homemade cakes |
|  |  | No waiter service |
|  |  | Sit down or takeaway |

| **4. Takeaway café/coffee, specialist and sandwich shop** | | |
| --- | --- | --- |
| Code | Type of food outlet | Description |
| 4.1 | Takeaway café | Predominantly coffee and hot beverages sold |
|  |  | No seating - takeaway only |
|  |  | Pre-made/ made to order sandwiches and confectionery available |
| 4.2 | Greasy spoon types café | Predominantly less healthy fried foods |
|  |  | No seating - takeaway only |
| 4.3 | Specialist café | Includes milkshakes/smoothie bars and ice cream shops |
|  |  | Similar in style to cafes and coffee shops |
|  |  | Takeaway only |
|  |  | Fair trade cafes/coffee shops are included here |
| 4.4 | Traditional sandwich shop | Made to order sandwiches/salads etc. |
|  |  | May sell drinks, branded snacks and homemade cakes |
|  |  | No sit in option - takeaway only |

| **5. Baker - retail** | | |
| --- | --- | --- |
| 5.1 | Baker - retail | Freshly baked savouries/bread, pre-made sandwiches, baked sweet products and branded products |
|  |  | Usually a chain e.g. Greggs, Milligan's, Bakers Oven but can be independent |

| **6. Takeaway and Fast food** | | |
| --- | --- | --- |
| Code | Type of food outlet | Description |
| 6.1 | Traditional takeaway | Hot food ordered and paid for at the till |
|  |  | Wait whilst food is prepared and cooked |
|  |  | No sit down option to eat-in but may have a seated waiting area |
|  |  | Usually open after 5pm |
| 6.2 | Traditional takeaway + delivery/collection | As traditional plus: The option to telephone for delivery and/or collection |
| 6.3 | Traditional takeaway + delivery/collection with seating | As traditional plus: Limited seating is available giving the option to eat-in |
|  |  | May have the option to telephone for delivery and/or collection |
| 6.4 | Instant fast food | Food ordered and paid for at the till |
|  |  | Available instantly as commonly cooked in bulk in advance and kept hot. Food that can be eaten without cutlery |
|  |  | Sit down, takeaway and drive-thru facilities |
|  |  | May be part of a chain or franchise |

| **7. Supermarket** | | |
| --- | --- | --- |
| 7.1 | Large multiple | Large, departmentalised, self-service food store selling food and household goods |
|  |  | E.g. Tesco, Asda, Morrisons, Sainsburys |
| 7.2 | Discount | E.g. Kwiksave, Netto, Lidl, Aldi |
| 7.3 | Small multiple | Smaller, self-service food store selling a limited range of food and household goods for greater convenience |
|  |  | E.g. Tesco metro/express, large Nisa/Premier |

| **8. Convenience** | | |
| --- | --- | --- |
| Code | Type of food outlet | Description |
| 8.1 | Traditional (corner shop) | Sells groceries, newspapers/magazines, snacks, drinks, lottery, tobacco products and sometimes pre-packed sandwiches |
|  |  | Usually have extended hours |
|  |  | Usually found in more residential areas |
| 8.2 | Newsagents | Small in size |
|  |  | Sells primarily newspapers, magazines, snacks, drinks and tobacco products |
|  |  | In well trafficked public places |
| 8.3 | Petrol station shop | Sells groceries, newspapers/magazines, snacks, drinks, lottery, tobacco products and sometimes pre-packed sandwiches |
|  |  | Usually have extended hours |
|  |  | May be a small multiple supermarket |
| 8.4 | Off-licence | Licensed to sell alcoholic beverages for consumption off the premises |
|  |  | Also sells groceries, newspapers, magazines, tobacco products, snacks and drinks |

| **9. Specialist (purchase to takeaway only)** | | |
| --- | --- | --- |
| Code | Type of food outlet | Description |
| 9.1 | Organic food stores |  |
| 9.2 | Health food stores | Health supplements |
|  |  | No fresh foods |
| 9.3 | Fair trade stores |  |
| 9.4 | Seasonal/ farmers market | Includes farmers markets and seasonal markets such as Christmas fayres |
| 9.5 | Artisan food stores | Stores selling only locally produced goods |
| 9.6 | Delicatessen | Grocery type store |
|  |  | Sells fresh ready-to-eat foods (made to order sandwiches/salads, cooked meats and cheeses etc.) |
| 9.7 | Wine merchant | E.g. Majestic, Oddbins |
| 9.8 | World food (all sizes) | E.g. oriental, Indian and continental shops and supermarkets |
| 9.9 | Candy/sweet/ chocolate shops | Shops that do not fall under the category of convenience or confectioners as sell only bought in sweets |
| 9.10 | Butcher | Fresh meat is prepared and sold in store |
| 9.11 | Baker | Bread and baked products prepared fresh and sold in store |
|  |  | Usually independent bakeries |
| 9.12 | Fishmonger | Fresh fish is prepared and sold in store |
| 9.13 | Greengrocer | Sells fresh fruit and vegetables |
| 9.14 | Dry goods only/weigh house | Dry good only, usually sold by weight |

| **10. Mobile food and market** | | |
| --- | --- | --- |
| Code | Type of food outlet | Description |
| 10.1 | Food provision | Food to take home |
|  |  | Usually needs preparation before consumption |
|  |  | E.g. meat stall at a farmers market |
| 10.2 | Takeaway food | Food to consume now |
|  |  | Usually found at outdoor events, e.g. music festivals, food matches, outside nightclubs |
| 10.3 | Ice cream van |  |
| 10.4 | Beverages | Includes coffee carts and smoothie stand |

**Appendix 4**

**Using Google Earth / Google Street View**

This instruction below provides tools for accessing Google Street View from Google Earth.

**Step one.** Open Google Earth

**Step two and three**. Enter the address of the street you want to audit in the search bar on the left side of your screen and drag Pegman to the starting point of the street segment


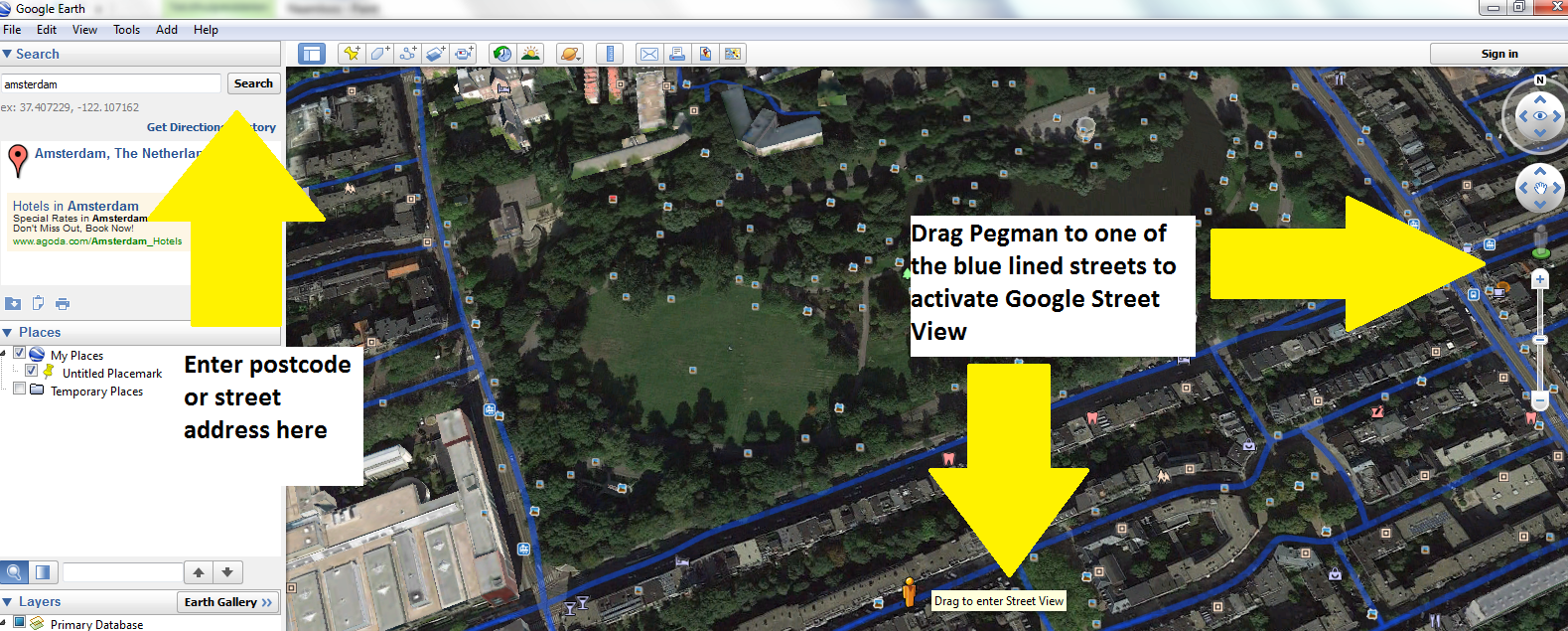


**Step four.** Enter Street View


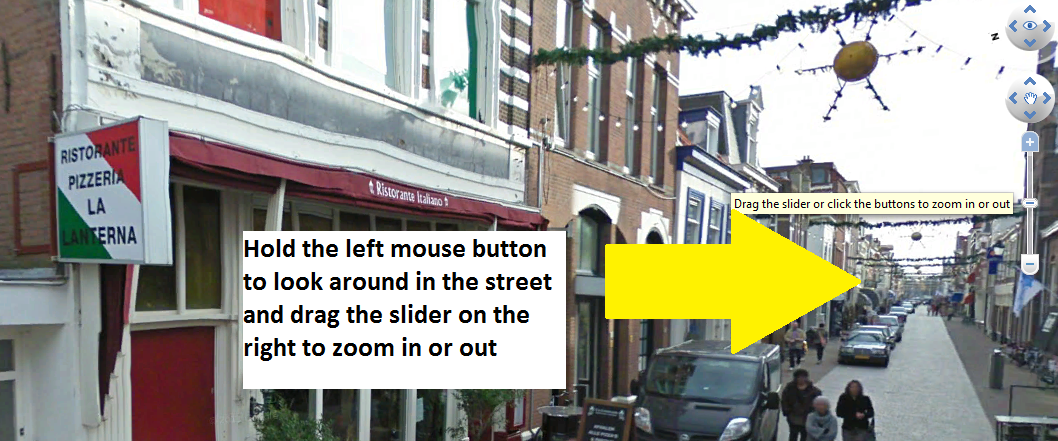


**Step five**. Tips and tricks


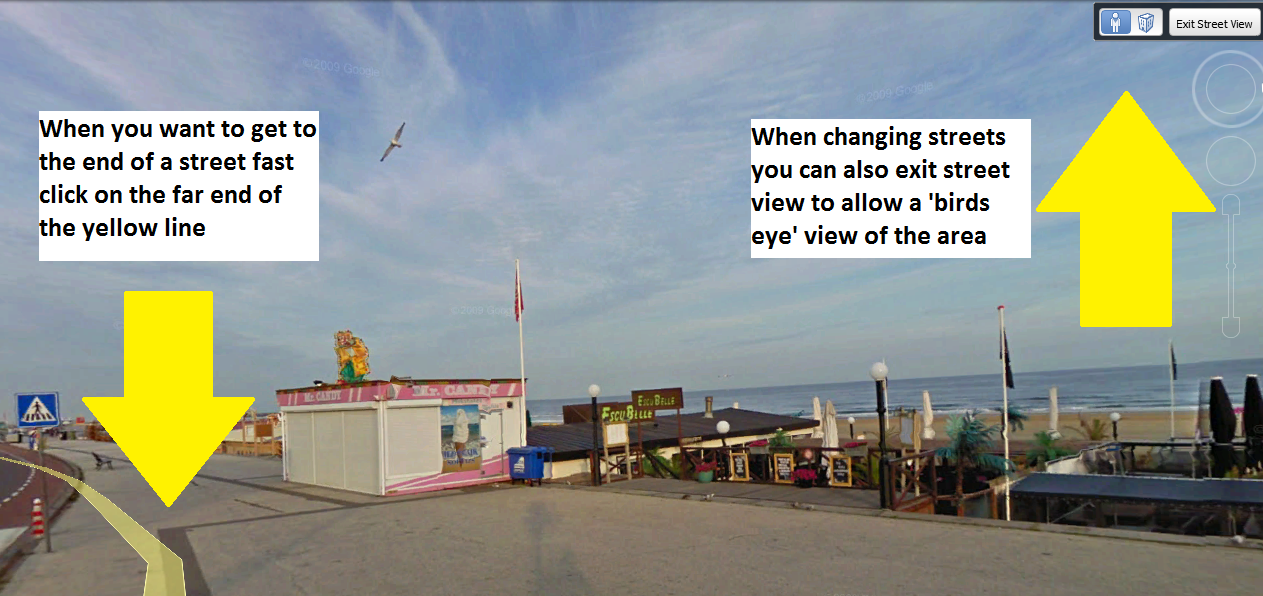

Supplement: Supplementary file 1 — Additional file 1: SOP Virtual Audit. Describes how the virtual audit is to be conducted. (DOC 13 MB) [file 12942_2014_615_MOESM1_ESM.doc]
