# Supplementary material for: The SPOTLIGHT virtual audit tool: a valid and reliable tool to assess obesogenic characteristics of the built environment
Source: Int J Health Geogr. 2014 Dec 16;13:52. doi: 10.1186/1476-072X-13-52 (PMC4279584; doi:10.1186/1476-072X-13-52)
Supplement: Supplementary file 2 — Additional file 2: SOP Field Audit. Describes how the field audit is to be conducted. (DOC 8 MB) [file 12942_2014_615_MOESM2_ESM.doc]

**Additional file 3**

**
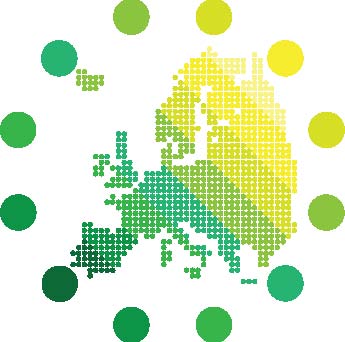
**

SPOTLIGHT

**The SPOTLIGHT field audit tool,**

**Standard Operation Procedure (SOP)**

**SPOTLIGHT WP3**: John Bethlehem, Helga Bardos, Johannes Brug, Helene Charreire, Sofie Compernolle, Ketevan Glonti, Jeroen Lakerveld, Joreintje Mackenbach, Jean-Michel Oppert, Maher Ben Rebah, Harry Rutter

**Content**

General Introduction 2

Preparation checklist and safety 3

Conducting the field audit 4

Definition of area and route 5

Saving of items 6

The SPOTLIGHT field audit tool 7

**General Introduction**

The SPOTLIGHT field audit tool (i.e. this SOP) is designed to assess key street-level features of the neighbourhood environment that are potentially related to physical activity and dietary behaviours - which are in turn related to obesity. SPOTLIGHT WP3 includes a sub-project which aims to assess actual environmental obesogenicity in selected neighbourhoods in five European Member States. Each Member State will individually assess their environmental obesogenicity. Therefore, the use of a standardised instrument will enable cross-country comparison of neighbourhoods for a common list of environmental characteristics. Environmental characteristics will be linked to health outcomes and lifestyle behaviours of inhabitants of the selected neighbourhoods.

The tool is based on a number of different existing tools (EPOCH 1, HAN, SPACES, ANC, PEDS, REAT, REIS, BESSC, Walkability audit tool and food environment classification tool). This tool assesses 8 general areas:

- Walking related items,
- Cycling related items,
- Public transport,
- Aesthetics,
- Land use-mix,
- Grocery stores,
- Food outlets,
- Physical activity facilities

Although much effort was put in this tool to be as comprehensive as possible, the tool is not complete in its assessment, which is an important aspect to bear in mind when analysing the results. Auditing neighbourhoods using this tool should be done by a researcher/research assistant who has a background in health-related research and has received training in the use of the tool. It is essential that researchers familiarise themselves with all components of the tool before assessing the streets.

In this protocol, brief instructions will be given on ways how to conduct field audits of neighbourhood characteristics, as well as operational definitions for key concepts or terms that are not self-explanatory in the tool.

**This SOP contains:**

- a preparation checklist of resources needed during the audit,
- a step by step protocol on how to conduct the field audit,
- definitions of the area and street segments and how to save the audited items
- a clarification of the included items,

**Preparation checklist and safety**

***Doing a street audit checklist:***

- Detailed map with pre-defined street segments per neighbourhood
- Printed copy of this SOP
- Clip board, paper, pencil
- Comfortable clothes & shoes
- Water bottle and snacks
- Cell phone with camera to capture special circumstances which could be of additional value to the audit.
- Cell phone with GPS function and/or GSV/GE
- Afterwards, transcribe data into field data entry file

**Safety**

Conduct the audit during daylight hours. If there is no safe place to walk, conduct audit from inside a vehicle or choose a safe vantage point. If there is no path or sidewalk, walk on the verge or edge of the road (if minimal to no traffic). If auditors feel threatened in any way, they should leave the area immediately and/or call police. If someone asks where you are from or what you are doing, and you feel comfortable responding, please respond with “from the *SPOTLIGHT project, mapping environmental characteristics*”. If someone further asks why you are doing this, please respond, e.g., “*to learn about the environmental factors that may influence our eating and physical activity behaviour*.” If someone continues to ask for more information, please respond “*for more information, you can call our project manager at {phone number}.*”

**Conducting the field audit**

1. Plan which street segments you are going to audit this session
2. Stand at the designated street segment according to your map
3. Get your pen and paper, audit tool, write down the segment/street ID and start auditing
4. Be sure to start at the correct side of the street segment so that you don’t audit in the wrong direction and can continue to the next street segment more easily when finished. You can be sure of this by checking which other streets that are visible on the intersection. Moreover, be sure that the street segment you are about to assess is the same as written down on your map
5. Start with auditing, on the left and right side of the street using the sequence given on the map
6. Familiarize yourself with the content of the tool, but if necessary, walk the segment twice or even three times to be sure you have filled out all the items
7. Clearly check boxes so no questions can be asked later as to which box is checked
8. Count buildings when necessary and write down ratings. In order to avoid double-counting of different types of buildings, buildings should be counted on the segment where the building’s address or main entrance is visible. A reference point should be noted on the continuous segment so that no duplication of information may occur
9. If needed, copy your answers to a neat version when returning to the university
10. After having rated the first segment, write down the second segment/street ID and follow the same procedure
11. Repeat this procedure until all segments of the street are assessed, before continuing to another street
12. When field audits are completed, the paper audits are transcribed into the field data entry file, which is comparable to the virtual audits. The SPOTLIGHT-VAT is modified to be used in field audits, however the content of the modified tool remains the same as the original virtual audit tool. With a little practice, the tool can be completed in approximately 5-10 minutes per street

**Definition of area, street segments and route**

*Choosing an area or route to audit*.

All streets are divided into street segments which have been given a unique number on the map and in the data entry file. It is therefore essential that each street is audited entirely (i.e. all noted street segments of the street) before starting to audit another street. It is recommended that the auditor starts at the top street on the map and works his/her way down, when starting to audit in each neighbourhood.

*Identifying Street Segments*.

Each street was virtually divided into street segments with a minimum length of 50 meters and a maximum length of 300 meters, between two intersections. This was done in order to maintain homogeneity of different street segments in long or short streets with few or many intersections. When segments were shorter than 50 meters but on a continuous part of the same street, segments were combined to one street segment. However, when segments are less than 50 metres and in a cul-de-sac, this segment was defined as a separate street segment. Streets were audited completely, or for a 300 additional meters (when streets are long and continue out of the audited area) when crossing the neighbourhood boundaries. Street segments were numbered so that different scored items can be better distinguished in the same street*.* Each street segment was given a unique code, usually starting with the first three or four letters of the street name and the sequence number of the street segment. This code was given in Google Earth and saved as a .kmz file for longitudinal studies in order to assess differences in environmental characteristics over time.

**Saving of items – technical details**

All paper versions of the field audits should be transcribed into the field data entry file. This needs to be done manually. In order to maintain a clear overview of which data is from the field audits a special data entry file is developed which is comparable to the virtual audits. It is crucial that each street segment is assessed with the same amount of concentration, so be sure to take a break when you are feeling a loss of concentration. Moreover, be certain that you only check one category per item and that it is clear which item is checked so transcription error can be prevented.

SPOTLIGHT Field audit checklist

| **Item** | **Category** | | | **Definition** | | |
| --- | --- | --- | --- | --- | --- | --- |
| **Walking related items** |  | | |  | | |
| Type of street: | Pedestrian friendly street | | | Area where motorized vehicles are allowed to drive max. 15 km/h. Road is shared with pedestrians, cyclists and cars. Children can play on the streets. This type of road can be an example of a 'woonerf' | | |
|  | Traffic sharing road | | | Road is mostly seen in residential or commercial area. Cars are allowed to drive, but need to give the right of way to cyclists and pedestrians. Maximum speed can differ | | |
|  | Regular road | | | Road in urban area where cars, cyclists and/or pedestrians have separated paths, with or without a buffer. Traffic speed does not exceed 50 km/h | | |
|  | Road with high-speed traffic | | | Main road that connects towns and cities, where speed exceeds 50 km/h. | | |
| Sidewalk present: | Yes/No | | | A man-made surface designed for pedestrians to use and can be only named sidewalk when it is associated with a roadway. | | |
| Sidewalk condition if sidewalk is present (see example 7) | Good | | | Good = no holes, sizable cracks, crumbling or uneven pavement on both sides of the street | | |
|  | Fair | | | Fair = holes, sizable cracks, or crumbling or uneven pavement along SOME of the side walk on both sides of the street | | |
|  | Poor | | | Poor = holes, sizable cracks, or crumbling or uneven pavement along most or ALL of the sidewalk on both sides of the street | | |
|  | Under construction | | | Under construction= (partially) blocked road, holes in road, construction work signage (only mark as under construction when there is a one sided sidewalk so that pedestrians have to walk on the road. When sidewalk on the other side of the street can be used, audit this part) | | |
| Pedestrian crossing available (see example 8) | Yes/No | | |  | | |
| Type of pedestrian crossing: | Zebra-path | | | Striped markings on the street, with or without signage for other road-users, but without traffic lights. | | |
|  | Over/underpass | | | Overpass is a bridge for pedestrians to cross over a street, underpass is a type of tunnel meant for pedestrians to cross under a street | | |
|  | Traffic lights | | | This can be any type of traffic lights meant for pedestrians to cross a street; automatic traffic lights, lights with pushbuttons etc. | | |
| Streetlights | Yes/No | | | This can be road- (for cyclists and cars) or pedestrian-oriented lighting and is present (at least two street lights) on a continuous stretch of road | | |
| 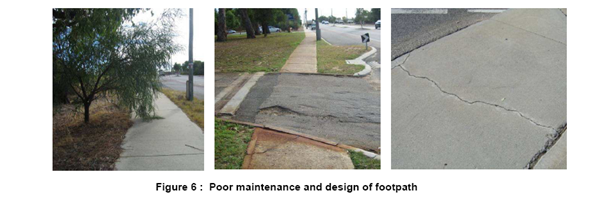  Example 7  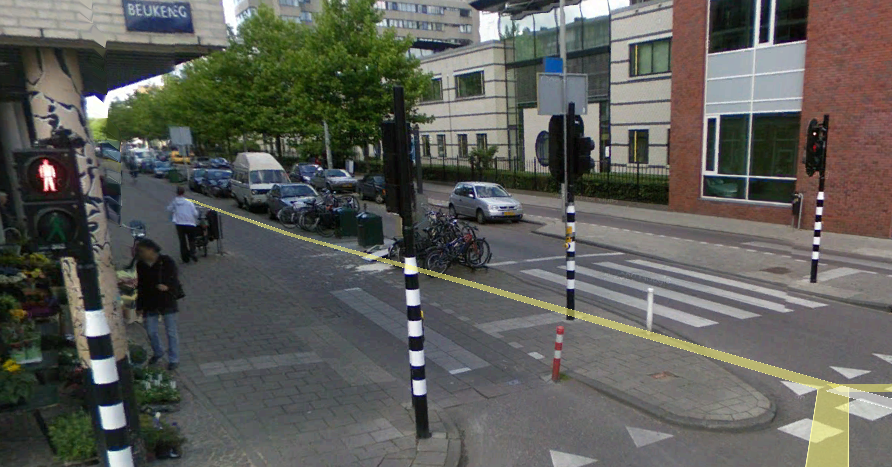  Example 8 | | | | | | |
| **Cycling related items** | | |  | |  | |
| Type of street: | | | Pedestrian friendly street | | Area where motorized vehicles are allowed to drive max. 15 km/h. Road is shared with pedestrians, cyclists and cars. Children can play on the streets. This type of road can be an example of a 'woonerf' | |
|  | | | Traffic sharing road | | Road is mostly seen in residential or commercial area. Cars are allowed to drive, but need to give the right of way to cyclists and pedestrians. Maximum speed can differ | |
|  | | | Regular road | | Road in urban area where cars, cyclists and/or pedestrians have separated paths, with or without a buffer. Traffic speed does not exceed 50 km/h | |
|  | | | Road with high-speed traffic | | Main road that connects towns and cities, where speed exceeds 50 km/h. | |
| Bicycle lane | | | Yes/No | | Lane on street where cyclists are allowed to cycle. This lane can or cannot be shared with other road-users | |
| What is the speed limit | | | Open space to write down speed | | Look at road speed signs on side of the street or on the road | |
| Obstacles present on bicycle lanes or place on road which is used by cyclists | | | Temporary | | This could be motorized vehicles, construction work, markets or any other objects that block the cycle path for a temporal time period. | |
|  | | | Permanent | | This could be a fence, building or any other obstacle which blocks the cycle path for a permanent time | |
|  | | | No | | No obstacles present which enables the cyclist to continue its path | |
| Cars form obstacles on road | | | Yes/No | | Cars parked on the road and/or partly on the sidewalk regardless of whether this is done legally or illegally. If cars are parked on the sidewalk and/or cycle path and cyclists and/or pedestrians have to manoeuvre around these cars, they form an obstacle | |
| Traffic calming devices | | | Yes/No | | Speed humps, traffic island, roundabouts, traffic lights | |
| Public bicycle facilities | | | Yes/No | | Facilities where bicycles can be rented. These facilities can be seen at for example railway-stations | |
| Type of bicycle lanes (see example 9) | | | On road cycle lane with markings | | Path used by cyclists, with centre line, logos and other markings included for safety or convenience | |
|  | | | Separate cycle lane with buffer | | Cycle lane is not shared with any other road-users and is separated from the street/sidewalk by a buffer and is specifically meant for cyclists | |
|  | | | Shared path with pedestrians | | Path used by pedestrians as well as cyclists, with or without markings | |
| 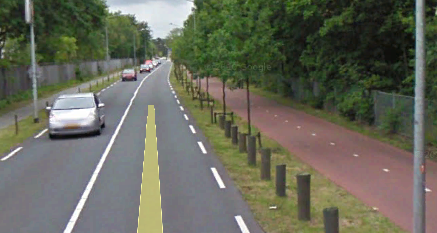 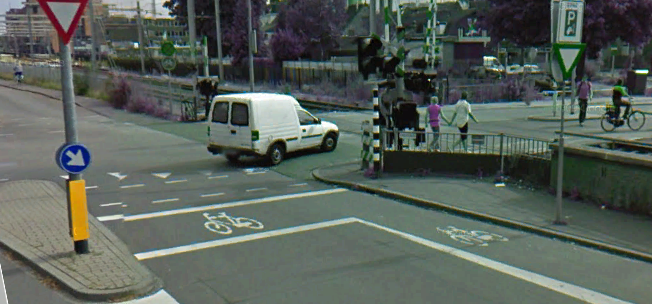  Separate cycle lane On road cycle lane with markings  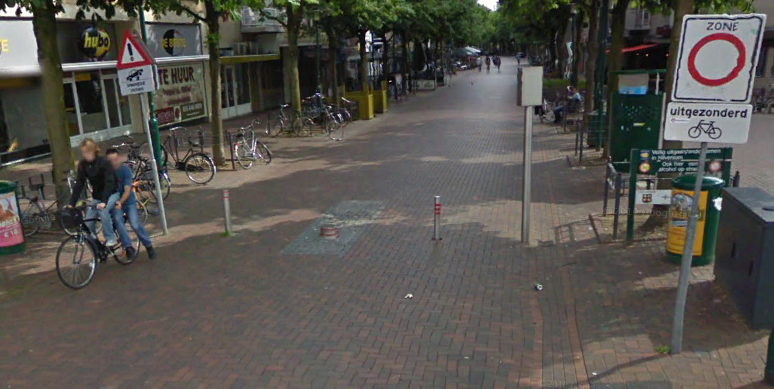  Shared path with pedestrians  Example 9   | **Public transport** |  |  | | --- | --- | --- | | Bus/tram stop | Yes/No | Presence of tram/bus transit stop where people have to wait in order to use the public transport facilities. Recognizable by signage, shelter or shoulder on road where bus/tram stops | | Railway/underground station | Yes/No | Presence of train/underground railway station. Recognizable by signage, train station | | | | | | | |
| **Aesthetics** | |  | | | |  |
| Green and water area visible | | Yes/No | | | | This can be any lake, river, park, forest or any other natural environmental surrounding |
| Residential gardens | | Yes/No | | | | Visibility of any residential gardens within street segment. If there is only a glimpse visible of a garden, then this garden would not be rated |
| Rating of condition of most residential buildings | | Well kept condition | | | | Housing looks well maintained, paint looks to be in good condition, gardens are taken care of |
|  | | Fair condition | | | | Housing is not well maintained but also not broken |
|  | | Poor/bad/detoriated condition | | | | Housing is not well maintained and broken windows, abandoned buildings are visible, gardens are not maintained |
| Abandoned building or vacant area | | Yes/No | | | | Building or area which is not being used. Only one vacant apartment should not be rated. When 50% or more of the apartment building is vacant then rate as abandoned. Only rate when it can be made clear that building houses no residents |
| Maintenance of green areas: | | Well maintained | | | | Verges, trees in residential gardens and/or public space, planted vegetation looks trimmed and clean |
|  | | Not well maintained | | | |  |
| Sidewalk condition if sidewalk is present (see example 7) | | Good | | | | Good = no holes, sizable cracks, crumbling or uneven pavement on both sides of the street |
|  | | Fair | | | | Fair = holes, sizable cracks, or crumbling or uneven pavement along SOME of the side walk on both sides of the street |
|  | | Poor | | | | Poor = holes, sizable cracks, or crumbling or uneven pavement along most or ALL of the sidewalk on both sides of the street |
|  | | Under construction | | | | Under construction= (partially) blocked road, holes in road, construction work signage (only mark as under construction when there is a one sided sidewalk so that pedestrians have to walk on the road. When sidewalk on the other side of the street can be used, audit this part) |
| Graffiti | | Yes/No | | | | Graffiti is defined as tags, and any other paint markings which could not be seen as street art which is placed on any buildings and or objects placed in the street |
| Litter | | Yes/No | | | | Trash visible on the streets, this can be a various range of discarded items like plastic bags, wrappings, cigarette packages and other discarded items laying on the street. Three or more discarded items on the streets can be scored as litter |
| Trees | | Yes/No | | | | Trees standing within this street segment |
|  | |  | | | |  |
| **Land use-mix** | |  | | | |  |
| Residential buildings visible | | Yes/No | | | | Housing where people only live in. Residential buildings need to be connected to the street where the audit is taking place. Front entrance or driveway must be connected to the street |
| Type of residential buildings: | | Detached/semidetached homes | | | | A house which is, or is not attached to another single house |
|  | | Terraced homes | | | | Multiple houses attached to each other |
|  | | Apartment buildings (<5 stories) | | | | Housing with apartments above and next to each other but the building is lower than (or equal to) 5 stories |
|  | | Apartment building (>5 stories) | | | | Housing with apartments above and next to each other but the building (including possible shops) is higher than 5 stories |
|  | | Apartment above shops | | | | Housing with one or more apartments above a shop |
| What is the percentage of non-residential buildings in comparison with residential buildings in this street segment | | 0% | | | | 0%= no residential buildings visible (rate 0% when there is only one or more non-residential buildings which in total are closer to 0% than 25%) , 25% = less non-residential buildings than residential buildings, 50% = about even distribution, 75%= more non-residential buildings than residential buildings, 100%= almost only non-residential buildings visible |
|  | | 25% | | | |  |
|  | | 50% | | | |  |
|  | | 75% | | | |  |
|  | | 100% | | | |  |
| **Grocery stores** | |  | | | |  |
| Supermarket | | Number and coordinates: | | | | Shop from an international or national chain where groceries/food supplies can be bought |
| Local food shop | | Number and coordinates: | | | | This can be a bakery, fish-shop, butcher, greengrocer |
| Street food market | | Number and coordinates: | | | | A vendor which sells food on a outdoor market. This can be fast-food or non-fast-food |
| Wine/liquor store | | Number and coordinates: | | | | Primarily sells alcohol containing drinks |
| Convenience store/small grocery store | | Number and coordinates: | | | | Smaller version of a supermarket but is from a local chain or from a gas-station company and can have different opening hours than larger supermarkets |
| **Food outlets** | |  | | | |  |
| Restaurant | | Number and coordinates: | | | | Non-fast-food restaurant, where people sit down to eat |
| Fast food restaurant | | Number and coordinates: | | | | This can be a international or local chain and is a destination that primarily sells carry-out burgers, fried chicken, sushi or other fast prepared foods (e.g. McDonalds, Döner kebab, KFC) |
| Take away restaurant | | Number and coordinates: | | | | Only rate when it is visible on the outside of the restaurant that this is a take away restaurant. This is the type of restaurant where people do not sit-down to eat but take their prepared meals elsewhere. See checklist food items for examples. |
| On-street vendors of food | | Number and coordinates: | | | | A vendor which sells fast-food on the street, like in a park or public square and sells hotdogs, spring-rolls or other snacks |
| Café/bar | | Number and coordinates: | | | | A place where people can drink coffee or other beverages with a small snack |
| Shopping mall | | Number and coordinates: | | | | Multiple shops housed in linked buildings |

| **Physical activity facilities** |  |  |
| --- | --- | --- |
| Indoor recreational facilities | Number and coordinates: | An indoor environment where people can exercise sports, or in any other way recreate. For example a gym, swimming pool or sports hall |
| Outdoor recreational facilities | Number and coordinates: | A man-made or natural outdoor environment where people can exercise sports, or in any other way recreate. For example a park, outdoor fitness area or skate park |
| Public park | Yes/No | Natural recreation area which is maintained and publicly accessible |
