# Supplementary material for: The SPOTLIGHT virtual audit tool: a valid and reliable tool to assess obesogenic characteristics of the built environment
Source: Int J Health Geogr. 2014 Dec 16;13:52. doi: 10.1186/1476-072X-13-52 (PMC4279584; doi:10.1186/1476-072X-13-52)
Supplement: Supplementary file 3 — Additional file 3: Table S1: Prevalence (%) of all SPOTLIGHT-VAT items per category, across different neighbourhood types and provides more detailed information on the study results. (DOC 120 KB) [file 12942_2014_615_MOESM3_ESM.doc]

**Supplementary Table 1**. Prevalence (%) of all SPOTLIGHT-VAT items per category, across different neighbourhood types

| **Category** | **HSES/HRAD** | **LSES/HRAD** | **HSES/LRAD** | **LSES/LRAD** | **Total** |
| --- | --- | --- | --- | --- | --- |
| **Walking related items** |  |  |  |  |  |
| *Type of street*:  Pedestrian friendly street  Traffic sharing road  Regular road  High speed traffic road | 1 (3.1%)  0 (0.0%)  31 (96.9%)  0 (0.0%) | 8 (40%)  0 (0.0%)  12 (60%)  0 (0.0%) | 1 (3.3%)  0 (0.0%)  29 (96.7%)  0 (0.0%) | 24 (52.2%)  0 (0.0%)  22 (47.8%)  0 (0.0%) | 34 (26.6%)  0 (0.0%)  94 (73.4%)  0 (0.0%) |
| Sidewalk present (% yes) | 31 (96.9%) | 14 (70%) | 16 (53.3%) | 22 (47.8%) | 83 (64.8%) |
| *Condition of sidewalk*:  Good  Fair  Poor  Under construction  Not present | 28 (87.6%)  1 (3.1%)  1 (3.1%)  1 (3.1%)  1 (3.1%) | 14 (70%)  0 (0.0%)  0 (0.0%)  0 (0.0%)  6 (30%) | 15 (50%)  1 (3.3%)  0 (0.0%)  0 (0.0%)  14 (46.7%) | 21 (45.6%)  0 (0.0%)  0 (0.0%)  1 (2.2%)  24 (52.2%) | 78 (60.9%)  2 (1.6%)  1 (0.8%)  2 (1.6%)  45 (35.1%) |
| Crossing available (% yes) | 4 (12.5%) | 1 (5%) | 0 (0%) | 3 (6.5%) | 8 (6.3%) |
| *Type of crossing*  Zebra path  Over/underpass  Traffic lights  Not present | 0 (0.0%)  0 (0.0%)  4 (12.5%)  28 (87.5%) | 0 (0.0%)  0 (0.0%)  1 (5%)  19 (95%) | 0 (0.0%)  0 (0.0%)  0 (0.0%)  30 (100%) | 3 (6.5%)  0 (0.0%)  0 (0.0%)  43 (93.5%) | 3 (2.3%)  0 (0.0%)  5 (3.9%)  120 (93.8%) |
| Streetlights (% yes) | 32 (100%) | 20 (100%) | 30 (100%) | 46 (100%) | 128 (100%) |
| **Cycling related items** |  |  |  |  |  |
| Bicycle lane (% yes) | 9 (28.1%) | 2 (10%) | 8 (26.7%) | 3 (6.5%) | 22 (17.2%) |
| Speed limit<50 km/h or 30 mph(% yes) | 20 (62.5%) | 18 (90%) | 16 (53.3%) | 43 (93.5%) | 97 (75.8%) |
| *Obstacles present on bicycle lanes*  Temporary  Permanent  No | 0 (0.0%)  2 (6.3%)  30 (93.7%) | 5 (25%)  2 (10%)  13 (65%) | 0 (0.0%)  0 (0.0%)  30 (100%) | 0 (0.0%)  1 (2.2%)  45 (97.8%) | 5 (3.9%)  5 (3.9%)  118 (92.2%) |
| Cars form obstacles (% yes) | 13 (40.6%) | 9 (45%) | 5 (16.7%) | 11 (23.9%) | 38 (29.7%) |
| Traffic calming devices (% yes) | 23 (71.9%) | 11 (55%) | 21 (70%) | 25 (54.3%) | 80 (62.5%) |
| Public bicycle renting facilities available (% yes) | 0 (0%) | 0 (0%) | 1 (3.3%) | 0 (0%) | 1 (0.8%) |
| *Type of bicycle lanes*  On road cycle lane with markings  Separate cycle lane with buffer  Shared path with pedestrians  Not present | 0 (0.0%)  9 (28.1%)  0 (0.0%)  23 (71.9%) | 2 (10%)  0 (0.0%)  0 (0.0%)  18 (90%) | 0 (0.0%)  8 (26.7%)  0 (0.0%)  22 (73.3%) | 2 (4.3%)  1 (2.2%)  0 (0.0%)  43 (93.5%) | 4 (3.1%)  18 (14.1%)  0 (0.0%)  106 (82.8%) |
| **Public transport** |  |  |  |  |  |
| Bus/tram stop present (% yes) | 1 (3.1%) | 2 (10%) | 3 (10%) | 3 (6.5%) | 9 (7%) |
| Railway/underground station present (% yes) | 0 (0%) | 0 (0.0%) | 1 (3.3%) | 0 (0.0%) | 1 (0.8%) |
| **Aesthetics** |  |  |  |  |  |
| Green/water area visible (% yes) | 12 (37.5%) | 1 (5%) | 12 (40%) | 23 (50%) | 48 (37.5%) |
| Residential gardens visible (% yes) | 9 (28.1%) | 3 (15%) | 30 (100%) | 35 (76.1%) | 77 (60.2%) |
| Rating of most residential buildings:  Well kept condition  Fair condition  Poor/detoriated condition  Not present | 23 (71.9%)  0 (0.0%)  0 (0.0%)  9 (28.1%) | 17 (85%)  0 (0.0%)  0 (0.0%)  3 (15%) | 29 (96.7%)  0 (0.0%)  0 (0.0%)  1 (3.3%) | 33 (71.7%)  0 (0.0%)  0 (0.0%)  13 (28.3%) | 102 (79.7%)  0 (0.0%)  0 (0.0%)  26 (20.3%) |
| Abandoned or vacant building/area visible: (% yes) | 6 (18.8%) | 1 (5%) | 3 (10%) | 0 (0%) | 10 (7.8%) |
| *Maintenance of green areas:*  Well maintained  Not well maintained  Not present | 25 (78.1%)  4 (12.5%)  3 (9.4%) | 17 (85%)  0 (0%)  3 (15%) | 29 (96.7%)  1 (3.3%)  0 (0%) | 46 (100%)  0 (0%)  0 (0%) | 117 (91.4%)  5 (3.9%)  6 (4.7%) |
| Graffiti present (% yes) | 10 (31.3%) | 1 (5%) | 4 (13.3%) | 0 (0%) | 15 (11.7%) |
| Litter on street/sidewalk (% yes) | 29 (90.6%) | 6 (30%) | 20 (66.7%) | 15 (32.6%) | 70 (54.7%) |
| Trees present (% yes) | 28 (87.5%) | 8 (40%) | 30 (100%) | 46(100%) | 66 (51.6%) |
| **Land use-mix** |  |  |  |  |  |
| Residential buildings visible from the street (% yes) | 23 (71.9%) | 17 (85%) | 29 (96.7%) | 33 (71.7%) | 102 (79.7%) |
| *Type of residential buildings:*  *Detached/semidetached homes*  Terraced homes  Apartment buildings <5 stories  Apartment buildings >5 stories  Apartment above shops  Not present | 0 (0.0%)  21 (65.7%)  0 (0.0%)  1 (3.1%)  1 (3.1%)  9 (28.1%) | 1 (5%)  12 (60%)  4 (20%)  0 (0.0%)  0 (0.0%)  3 (15%) | 28 (93.4%)  0 (0.0%)  1 (3.3%)  0 (0.0%)  0 (0.0%)  1 (3.3%) | 0 (0.0%)  29 (63%)  3 (6.5%)  1 (2.2%)  0 (0.0%)  13 (28.3%) | 29 (22.6%)  62 (48.4%)  8 (6.3%)  2 (1.6%)  1 (0.8%)  26 (20.3%) |
| *% of non-residential buildings*:  0%  25%  50%  75%  100% | 23 (71.9%)  2 (6.3%)  1 (3.1%)  1 (3.1%)  3 (9.4%)  *2 missing values* | 14 (70%)  2 (10%)  1 (5%)  1 (5%)  2 (10%) | 26 (86.7%)  2 (6.7%)  0 (0.0%)  1 (3.3%)  1 (3.3%) | 45 (97.8%)  0 (0.0%)  0 (0.0%)  0 (0.0%)  1 (2.2%) | 108 (84.4%)  6 (4.7%)  2 (1.6%)  3 (2.3%)  7 (5.5%) |
| **Grocery stores present** |  |  |  |  |  |
| Supermarket (% yes) | 0 (0.0%) | 0 (0.0%) | 1 (3.3%) | 1 (2.2%) | 2 (1.6%) |
| Local food shop (% yes) | 1 (3.1%) | 1 (5%) | 1 (3.3%) | 0 (0.0%) | 3 (2.3%) |
| Street food market (% yes) | 0 (0.0%) | 0 (0.0%) | 0 (0.0%) | 0 (0.0%) | 0 (0.0%) |
| Wine/liquor store (% yes) | 0 (0.0%) | 0 (0.0%) | 0 (0.0%) | 0 (0.0%) | 0 (0.0%) |
| Convenience store/small grocery store (% yes) | 0 (0.0%) | 1 (5%) | 0 (0.0%) | 0 (0.0%) | 1 (0.8%) |
| **Food outlets present** |  |  |  |  |  |
| Restaurant (% yes) | 0 (0.0%) | 0 (0.0%) | 0 (0.0%) | 0 (0.0%) | 0 (0.0%) |
| Fast food restaurant (% yes) | 1 (3.1%) | 0 (0.0%) | 0 (0.0%) | 0 (0.0%) | 1 (0.8%) |
| Take away restaurant (% yes) | 1 (3.1%) | 0 (0.0%) | 0 (0.0%) | 0 (0.0%) | 1 (0.8%) |
| On-street vendors of food (% yes) | 0 (0.0%) | 0 (0.0%) | 0 (0.0%) | 0 (0.0%) | 0 (0.0%) |
| Café/bar (% yes) | 1 (3.1%) | 0 (0.0%) | 0 (0.0%) | 0 (0.0%) | 1 (0.8%) |
| Shopping mall (% yes) | 0 (0.0%) | 0 (0.0%) | 0 (0.0%) | 0 (0.0%) | 0 (0.0%) |
| **Physical activity facilities present** |  |  |  |  |  |
| Indoor recreational facility(% yes) | 1 (3.1%) | 0 (0.0%) | 0 (0.0%) | 0 (0.0%) | 1 (0.8%) |
| Outdoor recreational facility (% yes) | 1 (3.1%) | 0 (0.0%) | 0 (0.0%) | 1 (2.2%) | 2 (1.6%) |
| Public park (% yes) | 2 (6.3%) | 0 (0.0%) | 2 (6.7%) | 2 (4.3%) | 6 (4.7%) |

SPOTLIGHT-VAT = SPOTLIGHT Virtual Audit Tool, HSES = high neighbourhood socio-economic status, LSES = low neighbourhood socio-economic status, HRAD = high urban residential density, LRAD = low urban residential density.
